# Thermal-Induced Percolation Phenomena and Elasticity of Highly Oriented Electrospun Conductive Nanofibrous Biocomposites for Tissue Engineering

**DOI:** 10.3390/ijms23158451

**Published:** 2022-07-30

**Authors:** Muhammad A. Munawar, Dirk W. Schubert

**Affiliations:** 1 Institute of Polymer Materials, Department of Material Science, Faculty of Engineering, Friedrich-Alexander-University Erlangen-Nuremberg, Martensstrasse 7, 91058 Erlangen, Germany; 2 KeyLab Advanced Fiber Technology, Bavarian Polymer Institute, Dr.-Mack-Strasse 77, 90762 Fürth, Germany

**Keywords:** nanofibrous biocomposites, dynamic percolation threshold, time-temperature superposition, Young’s modulus, biological tissues

## Abstract

Highly oriented electrospun conductive nanofibrous biocomposites (CNBs) of polylactic acid (PLA) and polyaniline (PANi) are fabricated using electrospinning. At the percolation threshold (*φ_c_*), the growth of continuous paths between PANi particles leads to a steep increase in the electrical conductivity of fibers, and the McLachlan equation is fitted to identify *φ_c_*. Annealing generates additional conductive channels, which lead to higher conductivity for dynamic percolation. For the first time, dynamic percolation is investigated for revealing time-temperature superposition in oriented conductive nanofibrous biocomposites. The crystallinity (*χ_c_*) displays a linear dependence on annealing temperature within the confined fiber of CNBs. The increase in crystallinity due to annealing also increases the Young’s modulus *E* of CNBs. The present study outlines a reliable approach to determining the conductivity and elasticity of nanofibers that are highly desirable for a wide range of biological tissue applications.

## 1. Introduction

Tissue engineering (TE) is an interdisciplinary field that combines stem cell biology, materials science, and engineering with the aim to generate constructs/scaffolds that resemble native human tissue. Modulation of scaffold’s properties such as; conductivity, elasticity, topography, geometry, and surface chemistry have a major impact on cell growth and adhesion/differentiation because cells receive different feedback from materials/scaffolds when they have the ability to dissipate the energy and reorganize their structures [1,2,3,4,5,6,7]. Tissues have complex structures, and, similar to engineering materials, their electrical, chemical, and mechanical properties are controlled by their microstructures [8]. Moreover, cell dynamics studies reveal that the morphology, orientation, proliferation, and migration of cells are affected by fiber orientation [9,10,11,12].

Highly oriented electrospun conductive nanofibrous biocomposites (CNBs) of biopolymers blending with electroconductive materials are the most suitable scaffolds for TE applications due to high orientation, tuneable conductivity, tuneable mechanical strength, tuneable fiber diameter, and biocompatibility [13,14,15]. The conductive components of CNB scaffolds develop intercellular signaling pathways between tissues and cultured cells, while non-conductive components provide a biocompatible environment to the cultured cells [1,6]. Electrospinning assists in the development of oriented CNBs with sufficient electrical, mechanical, and biological properties for effective tissue regeneration [16,17].

In our recent studies, electrical percolation thresholds (*φ_c_*) of CNBs of biopolymers (polyethylene oxide PEO, polycaprolactone PCL, and polylactic acid PLA) blended with intrinsically conductive polymers (ICPs)-fillers (Poly 3,4-ethylenedioxythiophene PEDOT and polyaniline PANi) were investigated in detail. The ICPs can form a percolating pathway for electrons and encourage the formation of electron-hole pairs, which can improve the electronic performance of oriented electrospun CNBs. Among conjugated polymers, PANi has become one of the most attractive ICPs because of its modulation of electrical conductivity by doping with organic acids such as camphorsulphonic acid (CSA). The chemical nature of ICPs (PANi, PEDOT, polypyrrole PPy) molecules remains mostly unchanged under annealing conditions, but the chain conformations can be altered substantially. These annealed-induced chain conformations of ICPs affect the mobility of charge carriers and thus control conductivity and percolation phenomena [18,19,20]. In ICPs-induced biopolymers, a conduction network is formed at the ICPs’ critical volume fraction/percolation threshold (*φ_c_*) depending on the structure/dispersion of ICPs and ICPs-matrix interactions and annealing conditions [21,22]. Annealing plays a vital role in controlling the conductivity of conductive electrospun fibrous composites. The conductive composites are in thermodynamic non-equilibrium. The formation of a conductive network is greatly dependent on annealing temperature *T* and time *t*. Annealing at higher temperatures can accelerate the structural evolution of the percolation network of filler, especially in a semicrystalline matrix, which is responsible for a drastic change in electrical resistance, termed dynamic percolation [23,24]. In a previous study, the CNBs of PEO, PCL, and PLA blended with doped PEDOT and PANi show that the percolation threshold decreases either with increasing annealing temperature *T* or time *t*, which leads to a hint of time-temperature superposition [21,22]. In this study, a detailed thermal-induced percolation phenomenon has been identified in CNBs of PLA-PANi fibers. For the first time, dynamic percolation is investigated for revealing time-temperature superposition in oriented electrospun conductive nanofibrous biocomposites using pre-existing polymer-based models. Additionally, the same fibers are investigated with respect to their mechanical properties. Therefore, fundamental work is now available, paving the way for biomedical applications of oriented conductive nanofibrous biocomposites.

Highly oriented CNBs of PLA (matrix) incorporated with doped PANi (filler) were fabricated using an electrospinning set-up deploying a proprietary designed rotating wheel collector, enabling high orientation. The electrical conductivity *σ*, percolation threshold *φ_c,_* and elasticity *E* of oriented CNBs were determined using our developed methods. The values of *φ_c_* were determined by fitting the McLachlan general effective media (GEM) equation to the experimental conductivities of CNBs. A linear relation between characteristics time *τ* and zero-shear viscosity *η_o_* was determined to prove time-temperature superposition in highly oriented CNBs. An exponential function with two adjustable fitting parameters was fitted to calculate the percolation threshold for infinite time of annealing *φ_c∞_* and characteristics time *τ*. A Carreau model was used to calculate the zero-shear viscosity *η_o_* of pure PLA matrix at different temperatures. Annealing semicrystalline polymers for long time periods at temperatures above their glass temperature *T_g_* and below their melting point *T_m_* has been shown as a method to increase the degree of crystallinity in the polymer [25]. The linear relationship between crystallinity *χ_c_* and annealing temperature *T* within confined fiber geometry was determined. In addition, the use of a semicrystalline matrix enables the ability to tailor the final mechanical properties of the composites by varying the degree of crystallinity. Moreover, a simple dependency of Young’s modulus/elasticity on crystallinity *χ_c_* of annealed CNBs has been revealed to have only two adjustable parameters. The morphology, alignment, and diameter of conductive fibers were determined using scanning electron microscopy (SEM) and transmission electron microscopy (TEM) analyses. The crystallinity *χ_c_* was calculated using differential scanning calorimetry (DSC) thermograms.

Since the deterioration of conductivity and elasticity of biological tissues has a profound effect on human morbidity and mortality [8,26], however, determining and correlating the conductivity *σ* and elasticity *E* of oriented conductive nanofibrous biocomposites (CNBs) is among the main motivations, to provide the right usage of conductive fibers for various biological tissue applications. Moreover, investigating the dynamic percolation is advantageous for optimizing annealing conditions to improve the electrical and mechanical performance of CNBs.

## 2. Results and Discussions

### 2.1. Aligned Conductive Nanofibers Using Electrospinning

The highly oriented electrospun CNBs of PLA-PANi:CSA were fabricated successfully using a special collector electrode (materials, preparation of the spinning solution, electrospinning set-up for oriented fibers, 3D-model of rotating wheel collector electrode, and process optimization have been stated in detail in Supporting Information in Sections S1–S5). At the optimized processing parameters, the spinning solution was ejected from the spinneret and stretched in the form of a charged jet toward the grounded collector. During the jet flight, stretching occurs due to electrical forces, while viscous forces counteract them, as discussed in [27] theoretically and revealed experimentally [28]. The displacement of jets from spinneret to collector and the evaporation (almost complete) of solvent led to solid CNBs on the rotating wheel electrode. A detailed electrospinning theory can be found in the works of Schubert [27,28]. The conductivity and kinematic viscosity of spinning solutions were determined and stated in Supporting Information in Section S6 and Section S7, respectively.

**Figure 1:**
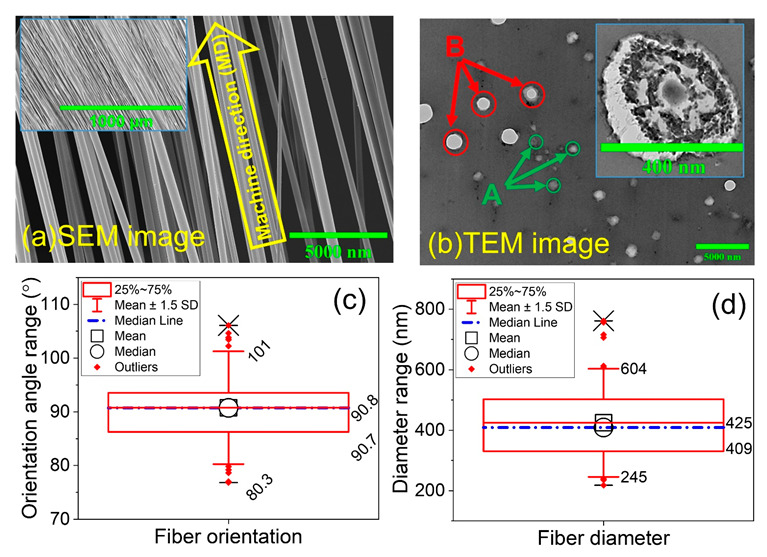
(**a**) shows the SEM images of aligned fibers having inset with lower magnification. (**b**) shows a cross-sectional TEM image of PLA-PANi:CSA fibers with *φ_PANi_* ≈ 7% concentration of filler; A: PLA-PANi:CSA fibers; B: artifacts from sample preparation. Inset shows a cross-sectional image of a single fiber. (**c**,**d**) show the directionality and diameter analysis with box plot analyses, respectively. The box outline is the interquartile range, the square and circle inside the box are the mean and median data points, the lines inside the box (red line and blue dash-dotted line) are the mean and median data locations, and the whiskers display the upper inner and lower inner fence values. Above and below the whiskers: (−) maximum/minimum data point and (×) 99th percentile.

The orientation and diameter of the fibers were measured with JMicroVision *v*.1.2.7 of 100 fibers on an SEM image with magnifications of 1000× and 5000×, respectively. The descriptive statistical analysis was carried out using Origin (OriginLab Corporation, USA, version 9.60) for constructing the box plots of orientation angle and fiber diameter. The SEM image (Figure 1a) shows that the fibers are straight and highly aligned in the machine direction MD, while the orientation angles of the fibers were measured against the vertical direction in the SEM image because the vertical axis of the image had been positioned to coincide with the MD. Figure 1b shows a TEM image of PLA-PANi:CSA fibers with *φ_PANi_* ≈ 7% concentration of filler. The different shades within the cross-section are possibly the phase structures/separation between PANi and PLA. From the box plot analysis (Figure 1c), it is evident that the fibers are predominantly oriented between 80° and 100°. The highest number of fibers was collected at an angle of 90°, spanning from 86° to 94°. Figure 1d shows a box plot analysis for fiber diameter with 409 nm and 425 nm mean and median diameter, respectively. The detail about SEM and TEM analyses have stated in Supporting Information in Section S8 and Section S9, respectively.

### 2.2. Percolation Phenomena in Oriented Conductive Nanofibrous Biocomposites

**Figure 2:**
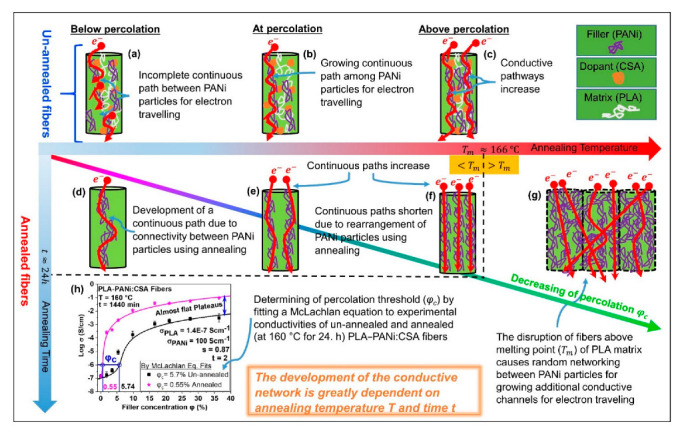
Schematic representation of percolation phenomenon within electrospun conductive nanofibers with and without annealing. (**a**) shows the incomplete continuous path between PANi (filler) particles (below the critical concentration/percolation threshold). (**b**) shows the completion of a continuous path between PANi particles for percolation phenomenon (at percolation threshold). (**c**) shows that continuous pathways increase with increasing filler concentration. (**d**) shows the development of a continuous path due to connectivity between PANi particles using annealing. (**e**) shows continuous paths doubling after annealing. (**f**) shows that continuous paths become double and shortened due to rearrangement of PANi particles at higher annealing temperatures. (**g**) shows annealing of conductive fibers above the melting temperature of PLA (T_m_ ~166 °C). The disruption of fibers above T_m_ of PLA matrix causes random networking between PANi particles for growing additional conductive channels for electron traveling. (**h**) shows the determination of percolation threshold (φ_c_) by fitting the McLachlan general effective media (GEM) equation to experimental conductivities of un-annealed and annealed (at 160 °C for 24 h) PLA-PANi:CSA fibers, respectively.

The schematic representation of percolation phenomena within conductive fibers of PLA-PANi:CSA is shown in Figure 2, where PLA and PANi act as matrix chains and filler particles, respectively. Below percolation (Figure 2a), there is no continuous path between PANi particles for electron traveling. At percolation (Figure 2b), the growth of the continuous path between PANi particles leads to a steep increase in the electrical conductivity of fibers. Above percolation (Figure 2c), continuous pathways increase until the conductivity of fibers becomes constant. During the annealing process, continuous paths between PANi particles develop below percolation (Figure 2d) and surge at and above percolation (Figure 2e,f). The annealing above the melting point of the PLA matrix (T_m_ > 166 °C) causes disruption of fibers and random networking between PANi particles, which leads to higher growth of additional conductive channels with a lowering percolation threshold (as schematically shown in Figure 2g). During the annealing process, the molecular chains of the PLA matrix become more relaxed, and PANi particles acquire a certain degree of freedom to interconnect/reorganize themselves. This reorganizing (intermingling) of ICPs generates more paths and additional conductive channels, which are responsible for higher conductivity with lower percolation threshold *φ_c_* [22]. The values of *φ_c_* were determined by fitting the McLachlan GEM equation to experimental conductivities of un-annealed and annealed PLA-PANi:CSA fibers. The percolation threshold of annealed PLA-PANi:CSA fibers at 160 °C for 24 h is *φ_c_* = 0.55%, which is one-tenth that of un-annealed fibers (*φ_c_* = 5.7%), as shown in Figure 2h.

The general effective media (GEM) equations (Equations (1a) and (1b) presented by McLachlan) [29] were used to determine the critical volume fraction/percolation threshold (*φ_c_*) of CNBs.

(1a) $$\left(1-\varphi \right)\;\cdot \;\frac{\sigma_{m}^{1/s}-\sigma^{1/s}}{\sigma_{m}^{1/s}+\left(\frac{1-\varphi_{c}}{\varphi_{c}}\right)\;\cdot \;\sigma^{1/s}}+\varphi \;\cdot \;\frac{\sigma_{f}^{1/t}-\sigma^{1/t}}{\sigma_{f}^{1/t}+\left(\frac{1-\varphi_{c}}{\varphi_{c}}\right)\;\cdot \;\sigma^{1/t}}=0$$

where *φ* is the volume fraction of filler in solid CNBs, *σ* is the electrical conductivity of CNBs, *σ_m,_* and *σ_f_* are the conductivities of pure PLA matrix and PANi filler, respectively (stated in Table S1), and *φ_c_* characterizes the percolation threshold. *s* and *t* are universal constants [30]. Critical exponents describe the behavior of physical quantities near continuous phase transitions. The exponents (*s* and *t*) are universal in the sense that they only depend on the type of percolation model and dimensionality in the system. They are expected to not depend on microscopic details such as the lattice structure or whether site/ bond percolation is considered. Notable is the finding that all data of the percolation curves can be fitted with an exponent *s* = 0.87 and *t* = 2 in the McLachlan equation [22]. In particular, *t* = 2 is most probably due to the fact that the percolation phenomena in nanofibers correspond to quasi-one-dimensional percolation phenomena, as the nanofiber is a confined geometry, and thus a one-dimensional percolation dominates.

To convert volume fraction (*φ*) to volume percent (*φ* %) of filler, Equation (1a) can be re-written as Equation (1b):

(1b) $$\left(100-\varphi \right)\cdot A+\varphi \cdot B=0$$

with:

$$A=\frac{\sigma_{m}^{1/s}-\sigma^{1/s}}{\sigma_{m}^{1/s}+\left(\frac{100-\varphi_{c}}{\varphi_{c}}\right)\;\cdot \;\sigma^{1/s}}\;\;\mathrm{and}\;B=\frac{\sigma_{f}^{1/t}-\sigma^{1/t}}{\sigma_{f}^{1/t}+\left(\frac{100-\varphi_{c}}{\varphi_{c}}\right)\;\cdot \;\sigma^{1/t}}$$

The volume fraction of filler *φ* in solid CNBs was calculated using Equation (2) (derived and stated under Supporting Information in Section S10).

(2) $$\varphi =\frac{xV_{fs}}{xV_{fs}+yV_{ms}}$$

where *V_ms_* and *V_fs_* are the volumes of matrix-solution and filler-solution, respectively.

The electrical conductivity *σ* of CNBs was calculated using Equation (3) (derived and stated under Supporting Information in Section S11).

(3) $$\sigma =\frac{\;L^{2}\;\cdot \;\rho_{c}}{R\;\cdot \;W_{c}}$$

where *L*, *ρ_c_*, *R,* and *W_c_* are length, density, resistance, and weight of CNBs, respectively.

The value of resistance *R* of CNBs on a glass slide (GS) was measured using a two-point probe method for a constant voltage (1 V) at room temperature. *W_c_* was measured before applying the silver-ink paste on GS, length *L* for all fibers is equal to the length of GS, and *ρ_c_* was calculated from Equation (4) (derived and stated under Supporting Information in Section S10).

(4) $$\rho_{c}=\frac{1}{\frac{WF_{f}}{\rho_{f}}+\frac{WF_{m}}{\rho_{m}}\;}$$

where *WF_f_* and *WF_m_* are the weight fractions of filler and matrix, while *ρ_f_* and *ρ_m_* are densities of filler and matrix, respectively.

### 2.3. Dependence of Percolation on Annealing Temperature and Time

**Figure 3:**
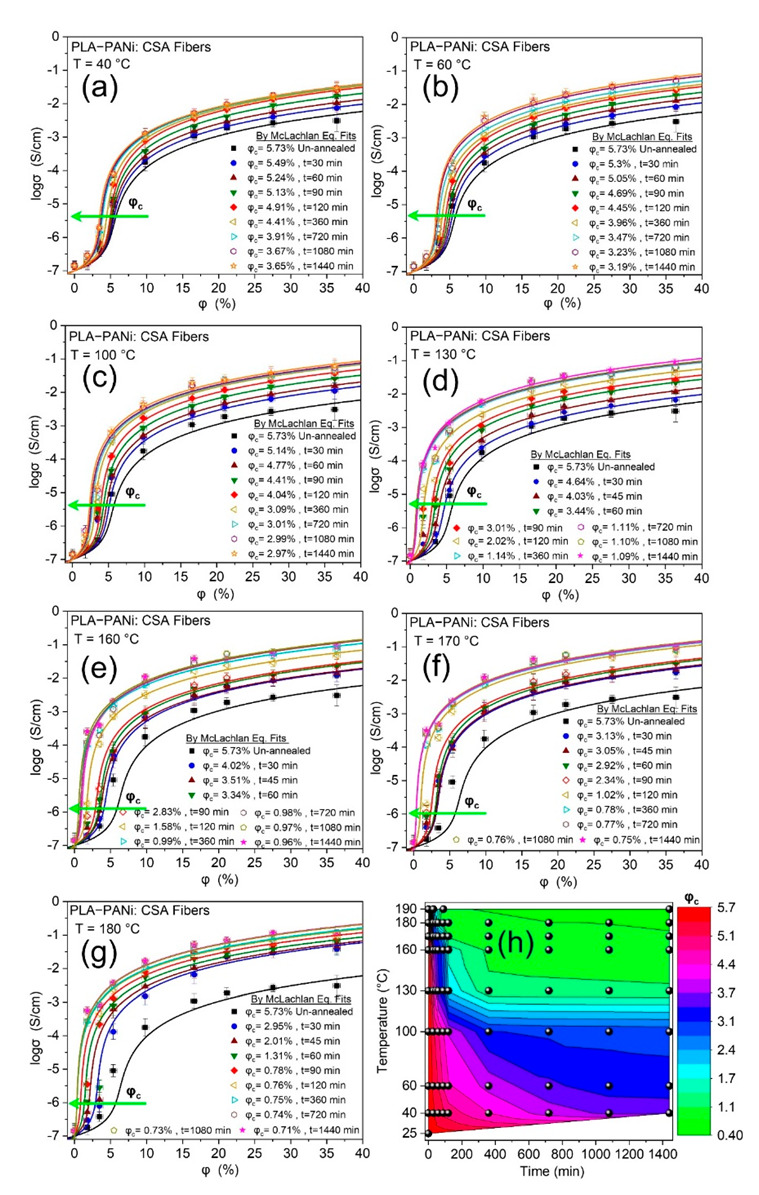
Logarithm of conductivity (*log σ*) against percentage volume fraction (*φ %*) of PANi for PLA-PANi:CSA fibers. (**a**–**g**) show the time-temperature dependence on percolation threshold *φ_c_*. The solid lines show the closest fits with the GEM model (Equation (1b)). (**h**) shows the contour plot representation between time, temperature, and *φ_c_*.

To check the annealing time-temperature dependence on percolation threshold *φ_c_* of PLA-PANi:CSA fibers (CNBs), conductive fibers were annealed at seven different temperatures (40, 60, 100, 130, 160, 170, and 180 °C) for eight time intervals (30, 60, 90, 120, 360, 720, 1080, and 1440 min). The details of the annealing process are given in the Supporting Information in Section S12. The resistance *R* of annealed CNBs is measured after quenching to room temperature. To ensure accuracy and precision, each sample of CNBs is fabricated at least three times. The conductivities of PLA-PANi:CSA fibers were determined, and all showed a steep increase in electrical conductivity on reaching a critical concentration, which corresponds to *φ_c._* Moreover, the GEM equation was fitted only when electrical conductivity reached almost flat plateaus to ensure a reliable fit. PANi was used as filler with an equal weight doping ratio to CSA (*PANi:CSA =* 1:1) with conductivity *σ_f_ =* 100 ± 5 S cm^−1^. PLA was used as a matrix, and the conductivity of pure matrix (*σ_m_*) for PLA fibers was determined at room temperature; *σ_m_ =* (1.39 ± 0.95) *×* 10^−7^ S cm^−1^. (The residual slovent is the reason for higher conductivity of pure PLA electrospun fibers as compared to literature). The maximum concentration of doped PANi, which was spun, was approximately 36%. Dynamic percolation was observed as a function of annealing temperature (*T*) and time (*t*). The GEM fitted curves and values of *φ_c_* for seven annealing temperatures of 40, 50, 60, 100, 130, 160, 170, and 180 °C and eight subsequent annealing times of 30, 60, 90, 120, 360, 720, 1080, and 1440 min are shown in Figure 3a–g, respectively, and the green arrows indicate the trend of decreasing *φ_c_*.

The motion of polymeric chains of a matrix under thermal relaxing is a significant factor for dynamic percolation threshold *φ_c_* in conductive nanofibrous composites. However, during the annealing process, the molecular chains of the host/matrix polymer become more relaxed, and filler particles acquire a certain degree of freedom to interconnect/reorganize themselves. This reorganizing (intermingling) of filler particles creates more paths and additional conductive channels, which are responsible for intra- and inter-chain conductivities in annealed conductive nanofibers [31,32]. Therefore, annealed CNBs can transport charge more efficiently for higher conductivity at a lower percolation threshold *φ_c_* [33,34,35,36].

Figure 3a–g show that the percolation threshold *φ_c_* of CNBs decreases at each annealing temperature with increasing annealing time and vice versa. At higher annealing temperatures, the formation of additional conductive channels is swift, which leads to lower *φ_c_* at very high rates and vice versa.

The conductive nanofibers are in a thermodynamic non-equilibrium state. However, the formation of conduction networks is dependent on temperature *T* and time *t*. The contour plot representation (Figure 3h) shows that either temperature *T* or time *t* increasing gives a lower percolation threshold, and vice versa. This leads to time-temperature superposition in conductive nanofibrous biocomposites CNBs, which is discussed in detail in the next section.

### 2.4. Percolation Reveals Time-Temperature Superposition in Conductive Nanofibers

An exponential function (Equation (5)) with two adjustable fitting parameters was used to calculate the percolation threshold for an infinite time of annealing (*φ_c∞_*) and a characteristic time (*τ*).

(5) $$\varphi_{c}=\left(\varphi_{co}-\varphi_{c\infty }\right)\;\cdot \;e^{-t/\tau }+\varphi_{c\infty }$$

where *φ_co_* is the initial value of the percolation threshold (*φ_co_*= 5.74) and *t* is time.

**Figure 4:**
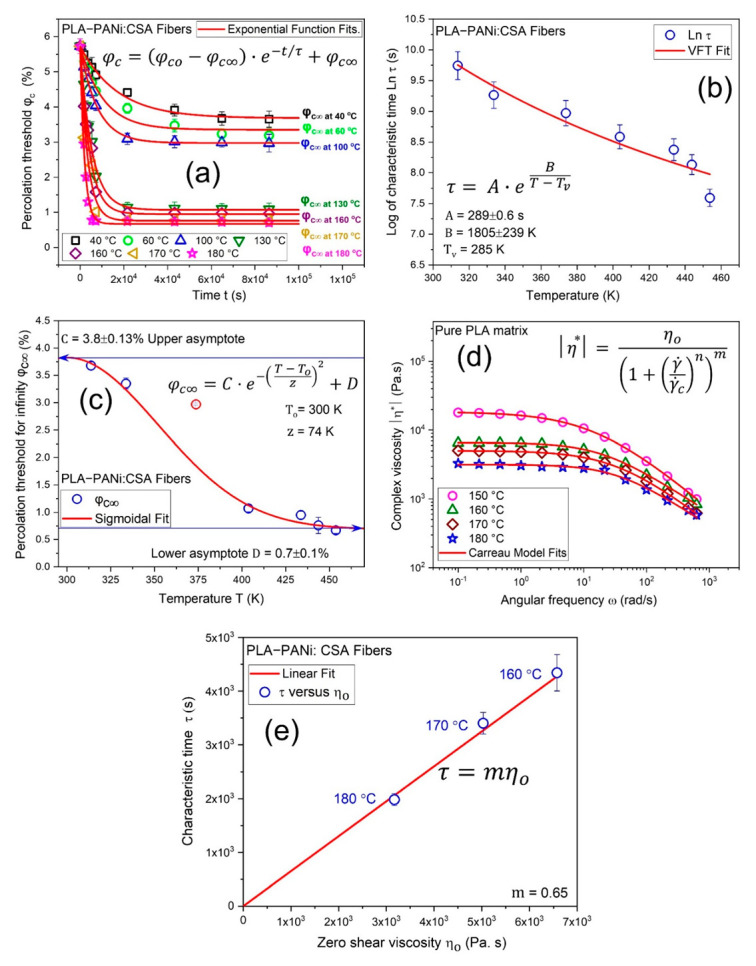
(**a**) shows an exponential function (Equation (5)) fit to determine infinite percolation threshold *φ_c∞_* and characteristic time *τ*. (**b**) shows a semi-logarithmic representation of the characteristic time *τ* as a function of temperature *T*, where the red solid line is a fit of the Vogel–Fulcher–Tammann (VFT) equation (Equation (6)). (**c**) shows a fit of sigmoidal function (Equation (7)) to describe *φ_c∞_* as a limit of temperature *T*. (**d**) shows the viscosity curve of PLA in relation to shear rate at 150, 160, 180, and 180 °C and a Carreau model (Equation (8)) is fitted to determine the zero-shear viscosity *η_o_*. (**e**) shows the linear dependence between characteristic time *τ* and zero-shear viscosity *η_o,_* and the linear fit is with intercept c = 0.

Figure 4a shows fitting curves of Equation (5) for determining *φ_c∞_* and *τ* at each annealing temperature (40–180 °C). Figure 4b shows a Vogel–Fulcher–Tammann (VFT) equation (Equation (6)) fit between the natural logarithm of characteristic time (*Ln τ*) and temperature (*T*) in Kelvin.

(6) $$\tau =A\;\cdot \;e{\;}^{\frac{B}{T-T_{v}}}$$

where *A* and *B* are fit parameters, and *T_v_* is also an empirical fitting parameter and typically lies 30−50 °C below the glass transition temperature *T_g_* of the polymer [37]. In this study, *T_v_* = 285 K was used because PLA has *T_g_* = 62 °C (335 K).

Figure 4c shows the sigmoidal fit function (Equation (7)) between *φ_c∞_* and temperature *T* with upper (*C* = 3.8%) and lower (*D* = 0.7%) asymptotes, while *T_o_* is the characteristic value of temperature and *z* describes the slope of fit function.

(7) $$\varphi_{c\infty }=C\;\cdot \;e^{-{\left(\;\frac{T-T_{o}}{z}\right)}^{2}}+D$$

The viscosities obtained from the frequency sweeps are fitted by a Carreau model (Equation (8)) to determine the zero-shear viscosity *η_o_* of pure PLA at different temperatures, as shown in Figure 4d.

(8) $$│\eta^{*}│=\frac{\eta_{o}}{{\left(1+{\left(\frac{\dot{\gamma }}{{\dot{\gamma }}_{c}}\right)}^{n}\right)}^{m}}$$

where $│\eta^{*}│$, $\dot{\gamma },$ and ${\dot{\gamma }}_{c}$ are complex viscosity, shear rate, and critical shear rate, respectively, and *m* and *n* are adjustable exponents. The experimental procedure used for determining the complex viscosity curve of pure PLA in relation to shear rate and temperature is given in Supporting Information in Section S13.

Figure 4e shows a characteristic time *τ* against zero-shear viscosity *η_o_* trend that is linear, which proves that percolation phenomena reveal time-temperature superposition in highly oriented conductive nanofibrous biocomposites CNBs. The hypothesis is that the annealing and the corresponding kinetics of the percolation composition are deformed by the viscosity of the semicrystalline polymer matrix, such that *τ ~ η_o_*. Therefore, a time-temperature superposition is evident.

### 2.5. Dependence of Crystallinity on Annealing within Confined Fiber Geometry

**Figure 5:**
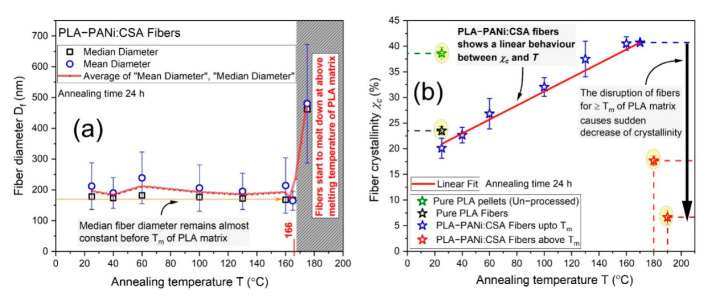
(**a**) shows the diameter analysis of PLA-PANi:CSA fibers at room temperature and 40, 60, 100, 130, 160, 165, and 175 °C, with *φ_PANi_* ≈ 10%. (**b**) shows the crystallinities (calculated from DSC data) of pure PLA pellets (unprocessed), pure PLA fibers, and PLA-PANi:CSA fibers at room temperature and 40, 60, 100, 130, 160, 170, 180, and 190 °C with *φ_PANi_* ≈ 10%. Solid red line shows the linear dependence between crystallinity *χ_c_* and annealing temperature *T* for PLA-PANi:CSA fibers.

Figure 5a shows the diameter analysis of PLA-PANi:CSA fibers for consecutive annealing temperatures up to and above the melting point of matrix polymer PLA. Moreover, at each annealing temperature (from 25 to 175 °C), the SEM image with its box plot analysis for median and mean fiber diameters was determined and shown in Supporting Information in Section S8. The median fiber diameter remains almost constant below the melting point of PLA. To study the dependence of the crystallinity on the temperature, differential scanning calorimetry (DSC) was used. The degree of crystallinity (*χ_c_*) was calculated using Equation (9) [38], and a detailed discussion of measurements and calculations of thermodynamic characteristics with DSC thermograms can be found in the Supporting Information in Section S14.

(9) $$\chi_{c}\left(\%\right)=\frac{\Delta H_{f}-\Delta H_{c}}{\Delta H_{f}^{⁰}}\;\cdot 100$$

where Δ*H_f_*_,_ Δ*H_c,_* and Δ*H_f_^⁰^* are the apparent heat of fusion per gram of the sample, enthalpy of cold crystallization, and the thermodynamic heat of fusion per gram of 100% crystalline polymer, respectively. The value of Δ*H_f_^⁰^* for PLA is 93 J/g [38].

Firstly, the crystallinity of unprocessed PLA pellets was higher than its electrospun fibers (as shown in Figure 5b). When the polymer solution is spun under high electrostatic forces (electrospinning process), the polymer solution is stretched and solidified rapidly while flying toward the collector. However, the polymeric chains do not have enough time to recrystallize again properly through the swift electrospinning process. Therefore, the oriented electrospun fibers always have a lower crystallinity than their unprocessed polymer (as shown in Figure 5b). Secondly, on incorporating the fillers, the crystallinity of PLA-PANi:CSA fibers decreased (as shown in Figure 5b), which was attributed to phase separation [39]. Thirdly, the crystallinity of oriented PLA-PANi:CSA fibers increased with annealing temperature, as shown in Figure 5b. There is a linear dependence between crystallinity *χ_c_* and annealing temperature *T* below the melting temperature (T_m_ = 166 °C) of PLA, considering the confined geometry of PLA-PANi:CSA fibers. The increase in crystallinity during annealing has a significant effect on the Young’s modulus/elasticity (*E*) of PLA-PANi:CSA fibers, as discussed briefly in the next Section 2.6. The final morphology of electrospun fibers after the annealing process depends on various factors, e.g., type of polymer, the molecular mass/molecular mass distributions, type of substrate, and type of filler [40]. Since PANi has a higher melting temperature (>350 °C) than PLA, annealing of PLA-PANi:CSA above the melting point (166 °C) of PLA promotes PANi particles to interconnect (reorganize) with each other to increase the conductivity.

### 2.6. Dependence of Fiber Elasticity on Filler Fraction and Annealing

The Young’s modulus (*E*) of a bundle of fibers can be determined using Equation (10) (derived and stated under Supporting Information in Section S15).

(10) $$E=k\;\cdot \;\frac{\rho_{c}\;\cdot \;L}{W_{c}}$$

where $k=\frac{F\;\cdot \;L}{\cdot L}$ is the slope of the force-strain curve in the linear elastic region, *ρ_c_*, *L*, and *W_c_* are the density, length, and weight of fibers in a bundle, respectively.

The crystallinity of electrospun fibers has a significant influence on mechanical properties, especially elasticity. However, the crystallinity of electrospun fibers can be altered by controlling molecular rearrangement, interaction, compatibility between filler particles and matrix chains, filler concentration, and subsequent annealing conditions within fiber geometry.

**Figure 6:**
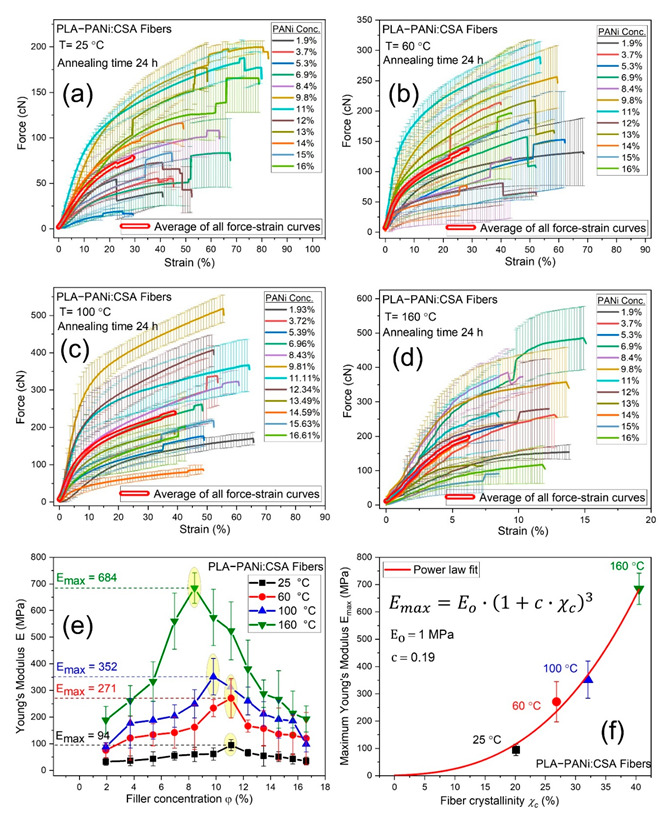
(**a**–**d**) show the force-strain curves for PLA-PANi:CSA fibers using 25, 60, 100, and 160 °C annealing temperatures, respectively. The percentage volume fraction (*φ %*) of PANi varies from 0% to 16%. The bold double red lines (in (**a**–**d**)) represent the average of all force-strain curves with common X range. (**e**) shows elastic Young’s modulus *E* as a function of percentage volume fraction (*φ %*) of PANi for PLA-PANi:CSA fibers at 25, 60, 100, and 160 °C annealing temperatures. (**f**) shows a power law correlation between maximum Young’s modulus *E_max_* and fiber crystallinity *χ_c_*.

Figure 6a–d show the force-strain curves of PLA-PANi:CSA fibers for various PANi concentrations (0–16 *φ* %) at room temperature (RT = 25 °C) and after annealing at 60, 100, and 160 °C for 24 h using a quenching process, respectively. Figure 6e shows the trending curves of elasticity *E* of PLA-PANi:CSA fibers at RT, 60, 100, and 160 °C as a function of *φ* % of PANi. The Young’s modulus *E* of PLA-PANi:CSA fibers increases to maximum Young’s modulus *E_max_* and then decreases as a function of *φ* % of PANi. The increase in elasticity *E* of un-annealed fibers (PLA-PANi:CSA) to an optimal concentration of filler PANi is due to the interactions of polymeric molecules (PLA matrix) with the surface of the filler particles, leading to restricted mobility of the attached molecules. The decrease in mechanical strength after the optimal concentration of PANi is expected due to the consequences of phase separation between molecular chains of PLA and filler PANi particles [41,42].

Semicrystalline polymers consist of highly oriented folded chain crystals connected by many highly stretched tie chains in the amorphous regions separating the crystals. Annealing changes the microstructure adopted by the polymer chains, and these changes strongly influence the mechanical properties of electrospun fibers [43]. On annealing, *E* also increases to *E_max_* then decreases as a function of *φ* % of PANi for each annealing temperature. The increase in elasticity *E* on annealing before the optimal concentration of PANi is anticipated due to an increase in the crystallinity of PLA-PANi:CSA fibers. The post-annealing process may cause some liberation of residual solvents and reaction between fillers, dopants, and matrices, which could liberate molecules (water/solvent) as by-products [22]. The loss of solvent reduces the weight of fibers, which increases the elasticity/stiffness of annealed nanofibers. Moreover, the residual solvents act as plasticizers, which are responsible for the low stiffness of un-annealed nanofibers. The removal of plasticizers (solvents) during annealing also increases the degree of crystallinity, which ultimately leads to higher elasticity in conductive fibers [21]. The annealing at concentrations above the optimal concentration of filler particles is also responsible for the initiation of crack growth behavior, which leads to lower mechanical strength of conductive fibers. However, annealing may also cause reactions between fillers, dopants, and polymer matrices, which could liberate (remove) various molecules (water/solvent) as by-products during the reaction [21,22]. The removal of residual solvent may also lead to some physical/chemical bonding among fillers and matrices, which are also responsible for increasing the dimensional stability. The maximum Young’s modulus *E_max_* has power law dependency on crystallinity *χ_c_* of annealed PLA-PANi:CSA fibers, as shown in Figure 6f, where a power law function (Equation (11)) was fitted between *E_max_* and *χ_c_*.

(11) $$E_{max}=E_{o}\cdot {\left(1+c\;\cdot \;\chi_{c}\right)}^{3}$$

where E_o_ is the value of the Young’s modulus when crystallinity *χ_c_* → 0 and exponent of 3 is motivated by a recent study [44].

### 2.7. Conductivity in Tissue Engineering

Electrical activity is a key feature of many types of tissues and organs such as skin, bone, cardiac, muscle, nerve, and cornea [17,24,45,46,47,48,49,50]. Conductive biocomposite scaffolds provide a promising technique for repairing various biological tissues. The conductivity of tissues (ventricular muscle, nerve, lung, cardiac, and skeletal muscle) lies typically between 10^−4^ and 10^−2^ S cm^−1^ [47,48,49]. An action potential can be produced artificially by changing the electrical potential of a nerve cell by inducing an electrical charge to the cells, and the process is termed ‘electrical stimulation’. The proposition related to electrical stimulation is based on the fact that bioelectricity present in the human body plays an integral role in maintaining normal biological functions, such as signaling of the nervous system, muscle contraction, and wound healing [50]. However, electrical stimulation as a physical stimulus draws much attention to tissue regeneration and its ability to influence cell migration, orientation, proliferation, and differentiation in tissue engineering [26]. Conductive nanofibrous biocomposite scaffolds have also been used to produce structures that mimic the mechanical properties of the extracellular matrix (ECM) of the heart muscle and improve the electrical signal propagation through scar tissue, potentially restoring cardiac functions. Aligned conductive fibers provide better cell attachment than random fibers and comparable electrical conductivity with superior mechanical properties [8,51,52].

**Figure 7:**
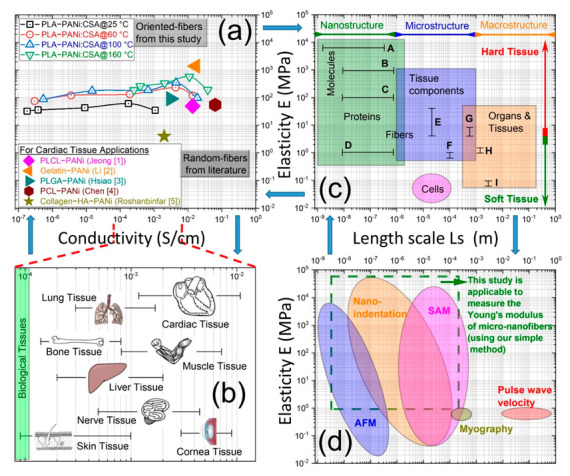
(**a**) shows a comparative study comparing the conductivities and Young’s moduli (elasticity) of our fabricated highly aligned conductive PLA-PANi:CSA fibers with other random conductive fibers, which have been reported in the literature for cardiac tissue engineering. (**b**) shows conductivity values of biological tissues, and values are expressed in S/cm [57]. (**c**) shows the length scale and elastic properties of soft tissues and their structural components. Superimposed are measurements of the elastic moduli of the aorta and ECM components at macroscopic, microscopic, and molecular length scales; A: single collagen fibrils [58], B: fibrillary collagen [59], C: fibrilin microfibrils [60], D: elastin [59], E. ferret aorta components [61], F: porcine aorta components [62], G: human radial artery [63], H: rat aorta [8], I: human aorta [64]. “Reproduced with permission Ref. [8] 2022, Riaz Akhtar” (**d**) schematic diagram illustrating the elastic modulus range and length scale of the typical spatial resolution attainable using a number of mechanical testing methods that have been successfully used with soft tissue samples. “Reproduced with permission Ref. [8] 2022, Riaz Akhtar”

Figure 7a shows a contrast/comparison of electrospun biocomposites of PANi-induced biopolymers for cardiac tissue applications. Our highly oriented conductive PLA-PANi:CSA fibers show superior conductivity and elasticity compared to random fibers reported in the literature. Moreover, Figure 7b shows that our conductive fibers can fulfill the electrical conductivity range of various biological tissues (10^−4^ – 10^−2^ S cm^−1^).

### 2.8. Elasticity in Tissue Engineering

Degradation and mechanical failure of tissues have a profound effect on human morbidity and mortality [8]. In biology, ECM is a dynamic non-cellular 3D structure in all tissues. The ECM consists of extracellular macromolecules and minerals, such as collagen, enzymes, glycoproteins, and hydroxyapatite, that provide structural and biochemical support to surrounding cells [53]. The stiffness/elasticity of the ECM has important implications because cells actively sense ECM rigidity, and it helps to regulate many important cellular processes, including cellular contraction, cell migration, cell proliferation, differentiation, and cell death [54]. The major structural protein in ECM is collagen having a rod-like fiber architecture [55]. However, electrospun fibrous scaffolds can mimic the ECM function for repairing/regenerating tissues [56].

Figure 7c shows a contrast between elastic stiffness of tissues and their structural components for the nano-macroscales. Nanoscale protein fibers have higher Young’s moduli than micro tissue components, which in turn are stiffer than the elasticity of macro tissue samples. The size and elasticity range for cells are also indicated for comparison. Our aligned conductive fibers have a Young’s modulus range of 20–700 MPa, which is comparable to the elasticity of most protein fibers and micro/macro tissue components/organs.

Figure 7d shows the comparison of different techniques used to measure the mechanical properties across the appropriate length scale of various tissues and their components. Nano-indentation and atomic force microscopy (AFM) techniques are better suited to measuring mechanical properties with micro-nanometre scale resolution of discrete tissue components. Scanning acoustic microscopy (SAM) is capable of mapping the mechanical properties of both discrete cells and whole tissues. Myography and pulse wave velocity (PWV) are used to estimate small and large artery stiffness, respectively. AFM, SAM, nano-indentation, myography, and PWV are expensive techniques and require great sensitivity during handling and testing of elasticity of electrospun fibrous scaffolds. This study shows that the elasticity of micro-nanofibrous scaffolds (fiber bundle on a few micro-millimeter scale) can be easily identified using the single-fiber tensile testing machine.

### 2.9. Biocompatibility of PANi-Based Conductive Fibers

Meanwhile, biocomposite is a category of biocompatible and/or eco-friendly (green) composites in a broad sense. However, based on the biocompatibility of PLA and PANi, it is referred to as biocomposites. In the literature, PANi-based nanostructured synthetic biopolymers (PCL, PLA, PVA and PEO) have proved their biocompatibility in various studies, as stated in Table 1. Wang et al. demonstrated that the PLA/PANi electrically conductive nanofibrous provide a conductive and biocompatible nanofibrous microenvironment for cardiomyocytes (CMs) viability, maturation, and synchronized beating [48]. Chen et al. determined that the incorporation of PANi into the PCL scaffolds does not induce significant toxicity to the myoblasts and also demonstrated that electrospun composite nanofibers can simultaneously provide topographical and electrical cues to cells, highlighting the importance of the synergistic effects for skeletal muscle tissue engineering [4].

With increasingly rigorous requirements for biomaterials, the design and fabrication of novel materials with smart functions are urgently needed. The fabrication of composite materials that can overcome individual shortcomings as well as bring synergistic benefits represents an efficient route to improve the performances and expand application scopes of biomaterials. Due to their unique structures and properties, electrospun fibers and hydrogels have been widely applied in many biological and biomedical fields. Based on this, more and more attention has been paid to the biocomposites of electrospun fibers and hydrogels as biomaterials, aiming to bring their individual supremacy into full play as well as rectify their intrinsic defects. However, in our next study, we will investigate the integration of highly oriented electrospun conductive nanofibrous biocomposites (CNBs) of ICPs in combination with hydrogels for tissue engineering applications.

## 3. Materials and Methods

### 3.1. Materials

PLA (Ingeo 4032D) containing 2% D-lactic acid and 98% L-lactic acid (Nature Works, Plymouth, MN, USA) and polyaniline (PANi) (Merck, Kenilworth, NJ, Germany) were used as polymer matrix and filler, respectively for fabrication of highly oriented electrospun conductive nanofibrous biocomposites (CNBs). Polyaniline emeraldine base (PANi-EB) was doped with (+)-Camphor-10-sulfonic acid (CSA) in equal weight ratio (*PANi:CSA* = 1:1). The characteristic properties of the materials used such as; molar mass (*Mw*), conductivity (*σ*) and values of melting point (*mp*) and density (*ρ*) have stated in detail in Supporting Information in Section S1. 

### 3.2. Methods

Electrospinning process was used to produce highly oriented electrospun conductive nanofibrous biocomposites of PLA and PANi. Figure S1 shows the schematics of preparation of spinning solution (Figure S1a), electrospinning set-up (Figure S1b–e) and methods for measurements of conductivity (Figure S1f–j) and elasticity (Figure S1k–q) of fibers and their details have stated in Supporting Information in Section S2, S3, S11 and S15, respectively. 

## 4. Conclusions

The highly oriented conductive nanofibrous biocomposites (CNBs) of PLA-PANi:CSA are in thermodynamic non-equilibrium, and the formation of conductive networks is highly dependent on annealing temperature and time. Annealing accelerates the structural evolution of the percolation network of filler (PANi) in the semicrystalline matrix (PLA), which is responsible for a drastic change in electrical conductivity, termed dynamic percolation. Investigating the dynamic percolation is advantageous for optimizing annealing conditions to improve the electrical and mechanical performance of fibrous biocomposites. The contour plot representation shows that either temperature or time increasing gives a lower percolation threshold. Percolation phenomena reveal time-temperature superposition in oriented electrospun CNBs of PLA-PANi:CSA based on the temperature dependence of the viscosity of the matrix polymer. The elasticity of PLA-PANi:CSA fibers increases up to optimal filler concentration. Prior to deterioration of matrix polymer PLA, the crystallinity of conductive nanofibers increases linearly with annealing temperature that leads to increasing the elasticity of annealed PLA-PANi:CSA fibers.

Moreover, the comparative study shows that our highly oriented electrospun conductive nanofibers have comparable/superior conductivity and elasticity compared to random fibers reported in the literature for various biological tissues and especially cardiac tissue engineering.

## Figures and Tables

**Table 1:** Polyaniline-based conductive constructs for tissue engineering.

Conductive Substrate	Mechanical Properties	Electrical Properties	Cell Line or Tissue	Biological Response
PLA/PANI electrospun membranes [48]	-	Four-probe technique, σ = 21 µS/m	H9c2, rat CMs	Myotube formation from H9c2 cells enhanced Cx43 and α-actinin expression improved Ca^2+^ transients for CMs
PCL, PANI electrospun membranes [4]	E = 55.2 MPa	Four-point probe, σ = 63.6 mS/cm	C2C12	Myotube formation
Chitosan, PANI patch [65]	E = 6.73 MPa	Four-probe technique, σ = 0.162 S/cm	Rat MI heart	Improved CV in the infarcted region with healing effects
PLCL, PANI electrospun membranes [1]	E = 50 MPa	Four-probe technique, σ = 13.8 mS/cm	Human fibroblasts, NIH-3T3, C2C12	Improved cell adhesion and metabolic activity
PU-AP/PCL porous scaffold [66]	E_c_ = 4.1 MPa	Four-probe technique, σ = 10^−5^ S/cm	Neonatal rat CMs	Enhanced Actn4, Cx43, and cTnT2 expressions
PANI/PCL patch [67]	-	Two-probe technique, σ = 80 µS/cm	hMSCs	Differentiation of hMSCs to CM-like cells
PDLA/PANI electrospun membranes [68]	-	σ = 44 mS/cm	Primary rat muscle cells	Improved cell adhesion and proliferation
Gelatin/PANI electrospun membranes [2]	E = 1384 MPa,	Four-probe technique, σ = 17 mS/cm	H9c2	Smooth muscle-like morphology rich in microfilaments
PLGA, PANI electrospun meshes [3]	E = 91.7 MPa	Four-point probe, σ = 3.1 mS/cm	Neonatal rat CMs	Enhanced Cx43 and cTnI expressions

## Supplementary Materials

The following are available online at https://www.mdpi.com/article/10.3390/ijms23158451/s1. References are cited in [69,70,71].
