# Supplementary material for: Thermal-Induced Percolation Phenomena and Elasticity of Highly Oriented Electrospun Conductive Nanofibrous Biocomposites for Tissue Engineering
Source: Int J Mol Sci. 2022 Jul 30;23(15):8451. doi: 10.3390/ijms23158451 (PMC9369359; doi:10.3390/ijms23158451)
Supplement: Supplementary file 1 [file ijms-23-08451-s001.zip › ijms-1827599-supplementary.pdf]

Supporting Information

Thermal-induced Percolation Phenomena and Elasticity of Highly Oriented Electrospun Conductive Nanofibrous Biocomposites for Tissue Engineering

Muhammad A. Munawar [1,2] and Dirk W. Schubert [1,2]**

[1] Institute of Polymer Materials, Department of Material Science, Faculty of Engineering, Friedrich-Alexander-University Erlangen-Nuremberg, Martensstrasse 7, 91058 Erlangen, Germany

[2] KeyLab Advanced Fiber Technology, Bavarian Polymer Institute, Dr.-Mack-Strasse 77, 90762 Fürth, Germany

***** Correspondence: muhammad.munawar@fau.de (M.A.M.); dirk.schubert@fau.de (D.W.S.)

S1. Materials

PLA (Ingeo 4032D) containing 2% D-lactic acid and 98% L-lactic acid (Nature Works, USA) and polyaniline (PANi) (Merck, Germany) were used as polymer matrix and filler, respectively for fabrication of highly oriented electrospun conductive nanofibrous biocomposites (CNBs). The electrical conductivity of pure polymer matrix (σ_m) was measured using the electrospun fibers of pure PLA on glass slide (value is shown in **Table S1** and a bit higher value is due to glass slide substrate and also some moisture effect). Polyaniline emeraldine base (PANi-EB) was doped with (+)-Camphor-10-sulfonic acid (CSA) in equal weight ratio (PANi:CSA = 1:1) and the electrical conductivity of doped-PANi (σ_f) is stated in **Table S1**. The solvents ethanol (Et-OH) denatured ≥ 99.8, N,N-dimethyl formamide (DMF) ≥ 99.8 and chloroform (CF) were purchased from Merck (Sigma-Aldrich) Germany. The characteristic properties of the materials used such as; molar mass (*Mw*), conductivity (σ) and values of melting point (*mp*), boiling point (*bp*), density (ρ) are shown in **Table S1**.

Table S1. Characteristics and properties of materials used.

Materials	Molar mass Mw	Melting Point mp	Density ρ at 25˚C	Conductivity σ
	(kDa)	(˚C)	(g cm^{-3})	(S cm^{-1})
PLA	109	166	1.24	≈ 1.39 ± 0.9)×10^{-7} (PLA fibers on GS)
PANi-EB	65	> 350	1.101	≈ (1.1)×10^{-9} (pressed pellet, ASTM F8).<br>PANi:CSA (1:1) in weight ratio; ≈ (100 ± 5)
CSA	232.30 Da	200	1.302	-
H_2O	18.02 Da	-	1.000 at 3.98 ˚C	≤ (0.05)×10^{-6}
CF	119.38 Da	-	1.48	≤ (0.02)×10^{-6}
Et-OH	46.07 Da	-	0.816	Non-conductive
DMF	73.09 Da	-	0.944	≤ (0.3)×10^{-6}

S2. Preparation of spinning solution

The schematic procedure for preparation of spinning solutions is shown in **Figure S1a**. The matrix-solution and filler-solution/dispersion with their respective solvent systems were prepared separately, via step 1 and step 2, respectively. The independently prepared matrix-solution and filler-solution were mixed in different volume ratios (step 3). The procedure for

the derivation of equations for calculating the volume fraction of filler (φ) and density (ρ_c) of electrospun conductive nanofibrous biocomposites (CNBs) are explained in Section S10.

S3. Electrospinning set-up for oriented fibers

The electrospinning set-up (schematic shown in **Figure S1b-d**) consists of three main parts: a syringe with feeding pump delivering the spinning solution, a high voltage (HV) power supply (0-60 kV) and a rotating collector for the fibers, which was purpose-built at the University of Erlangen-Nuremberg. The fibers collected on the rotating wheel are straight and highly aligned in the machine direction MD, as shown in the photo (**Figure S1e**) of the wheel with fibers on it. The rotating collector consists of two circular terminal plates equipped with circular notches. The notches enable mounting of equally spaced horizontal bars. The bars are held in place by screws. In this study, sixteen bars were used. The spacing between two adjacent bars is 25 mm (2.5 cm). The rotating collector has a diameter of 21.2 cm. It can be used up to a speed of 1000 rpm, which corresponds to a tangential velocity of approximately 11 m/s. The matrix-filler solutions were poured into a 10 ml glass syringe with a blunt 20-gauge stainless steel nozzle with an inner diameter of approximately 0.61 mm and a flat tip. The conductivity and kinematic viscosity of spinning solutions were determined and stated in in Section S6 and Section S7, respectively. The high voltage power supply (60 kV, DC Linari Engineering S.r.l., and Valpiana, Italy) was used to produce a direct current with the positive polarity on the metallic nozzle while the collector was grounded. A feeding pump (Linari Engineering S.r.l., and Valpiana, Italy) was used to provide a constant flow-rate of the spinning solution. The processing parameters were varied and their ranges were; voltage (15-25kV), flow-rate (0.5-1.5 mL/h), tip-collector distance (9-15 cm) and collector rotation (100-400 rpm). At the optimized processing parameters, the spinning solution was ejected from the spinneret and stretched in the form of a charged jet towards the grounded collector. The optimization of processing paramters has been stated in detail in Section S5.

During the jet flight, stretching occurs due to electrical forces, while viscous forces counter act them as discussed in [1] theoretically and revealed experimentally [2]. The displacement of jets from spinneret to collector and the evaporation (almost complete) of solvent led to solid CNBs on the rotating wheel electrode. A detailed electrospinning theory can be found in the works by Schubert [1,2].

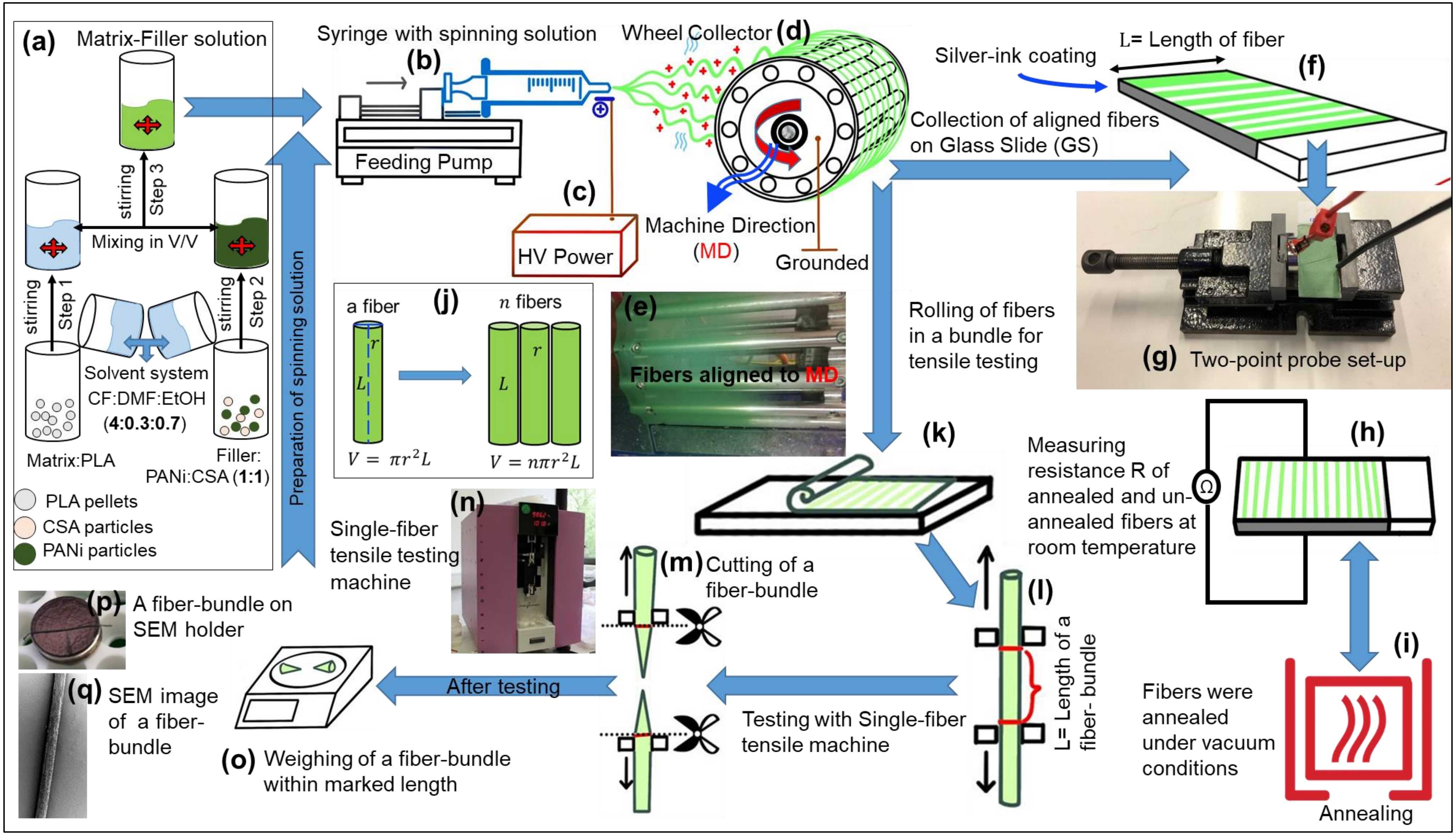
(a) Matrix-Filler solution
stirring
Step 3
Mixing in V/V
stirring
Step 1
stirring
Step 2
Solvent system
CF:DMF:EtOH
(4:0.3:0.7)
Matrix:PLA
Filler:
PANi:CSA (1:1)
PLA pellets
CSA particles
PANi particles
Preparation of spinning solution
Syringe with spinning solution
(b)
Feeding Pump
(c)
HV Power
Wheel Collector (d)
Machine Direction
(MD)
Grounded
Silver-ink coating
L= Length of fiber
(f)
Collection of aligned fibers
on Glass Slide (GS)
(g) Two-point probe set-up
a fiber
(j)
n fibers
$V = \pi r^2 L$
$V = n\pi r^2 L$
(e)
Fibers aligned to MD
Rolling of fibers
in a bundle for
tensile testing
(k)
Measuring
resistance R of
annealed and un-
annealed fibers at
room temperature
(h)
Ω
(n)
Single-fiber
tensile testing
machine
(m) Cutting of a
fiber-bundle
(l)
L= Length of a
fiber- bundle
(i)
Fibers were
annealed
under vacuum
conditions
Annealing
(p) A fiber-bundle on
SEM holder
(q) SEM image
of a fiber-
bundle
After testing
Testing with Single-fiber
tensile machine
(o) Weighing of a fiber-bundle
within marked length

Figure S1. Schematic of preparation of spinning solution, electrospinning set-up and procedure for measurements of conductivity and elasticity of CNBs. Panel a shows the steps for preparation of matrix-filler solution. Panels b, c and d show the feeding pump, high voltage (HV) power supply and rotating wheel collector, respectively. The wheel covered with electrospun fibers aligned to machine direction (MD) is also shown in photo (panel e). Panels f, g, h and i show the glass slide (GS) with aligned fibers of length L, two-point probe set-up, Ohmmeter for resistance R measurement and annealing process, respectively. Panel j shows the schematic of a single fiber of length L and radius r and n fibers. Panels k, l and m show the rolling of fibers into a bundle, clamping of a fiber-bundle (within red marks of length L) and testing of a fiber-bundle, respectively using a single-fiber tensile testing machine (Panel n). Panel o shows weighing of the tested sample within the marked length. Panels p and q show a fiber-bundle on SEM holder and SEM image of a fiber-bundle, respectively.

S4. 3D-model of rotating wheel collector electrode

A 3D model and its geometrical diagram are shown in **Figure S2** and **S3**, respectively

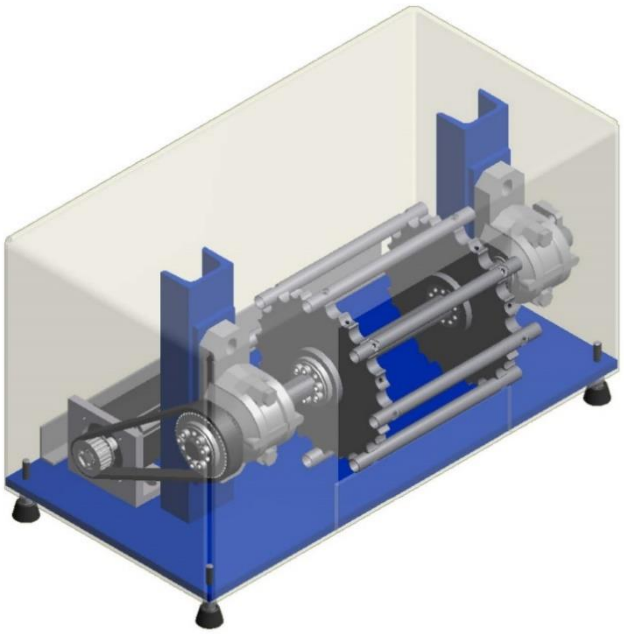

Figure S2. Custom made 3D-model of rotating wheel collector electrode, driven by a v-belt.

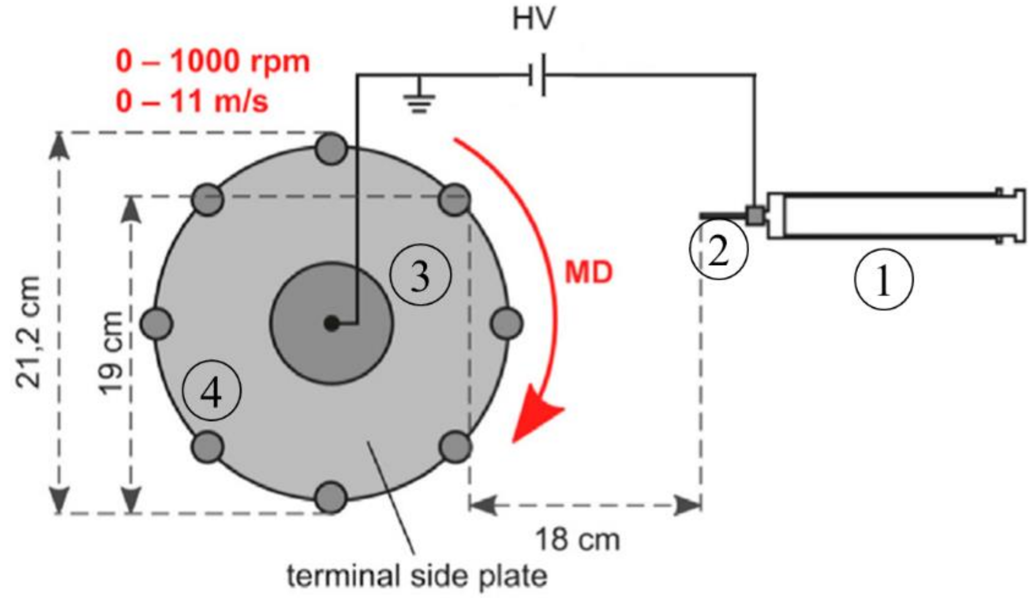

Figure S3. Geometrical diagram of rotating wheel collector electrode where;

1. Syringe with solution
2. Needle (Anode)
3. Rotating collector (Cathode)
4. Metallic circular bars for collecting fibers

S5. Optimization of electrospinning processing parameters

The processing parameters were optimized regarding based on fiber diameter for PLA (matrix) and then quantified for PLA/PANi spinning solutions with subsequent increasing of PANi (filler) concentration. Among one of our case studies, electrospinning process was used to produce highly oriented electrospun fibers of PLA utilizing a special rotating collector electrode. Corresponding fiber diameter was analysed, varying the process parameters according to our emperical scaling laws (**Equation S1**) for polymer solution concentration (c), viscosity (η), flow-rate (Q), spinning-distance (X), voltage (U), electric current (I) and rotation-speed of the collector (ω).

$$d_f = k \cdot \left(\frac{\sqrt{c} \cdot \eta^{\frac{1}{3}} \cdot Q^{\frac{1}{5}} \cdot X^{\frac{2}{3}}}{U^{\frac{2}{3}} \cdot \omega^{\frac{1}{4}} \cdot I^{\frac{1}{5}}} \right) \qquad \text{(S1)}$$

Where k = 1674 nm for this specified system (PLA solution) taking solution conductivity (σ) and surface tension (γ) as constant. For increasing the PANi concentration, the fiber diameter varies according $D \sim \sigma^{-2/3}$. Moreover, the qualitative and quantitative validation of **Equation**

S1 is shown in **Figure S4** using the optimized processing paramters as stated in **Table S2**. This part of work is in under publication process in our study with the title: ‘Modeling and optimization of diameter of highly oriented electrospun nanofibers of biopolymer’ [3]. However, in the latest studies, Schubert has demonstrated theoretically and experimentally, the novel scaling laws for various processing paramters in detail.

Table S2. Range of optimized processing paramters.

**Concentration**	**Viscosity**	**Flow rate**	**Voltage**	**Distance**	**Rotation**
c	η	Q	U	X	ω
(%)	(mm^2/s)	(mL/h)	(kV)	(cm)	(rpm)
9	193	0.5	25	9	400
9	193	1	25	12	400
9	193	1.5	25	15	400
9	193	0.5	20	9	200
9	193	1	20	12	200
9	193	1.5	20	15	200
9	193	0.5	15	9	100
9	193	1	15	12	100
9	193	1.5	15	15	100
10	292	0.5	25	9	400
10	292	1	25	12	400
10	292	1.5	25	15	400
10	292	0.5	20	9	200
10	292	1	20	12	200
10	292	1.5	20	15	200
10	292	0.5	15	9	100
10	292	1	15	12	100
10	292	1.5	15	15	100
11	414	0.5	25	9	400
11	414	1	25	12	400
11	414	1.5	25	15	400
11	414	0.5	20	9	200
11	414	1	20	12	200
11	414	1.5	20	15	200
11	414	0.5	15	9	100
11	414	1	15	12	100
11	414	1.5	15	15	100

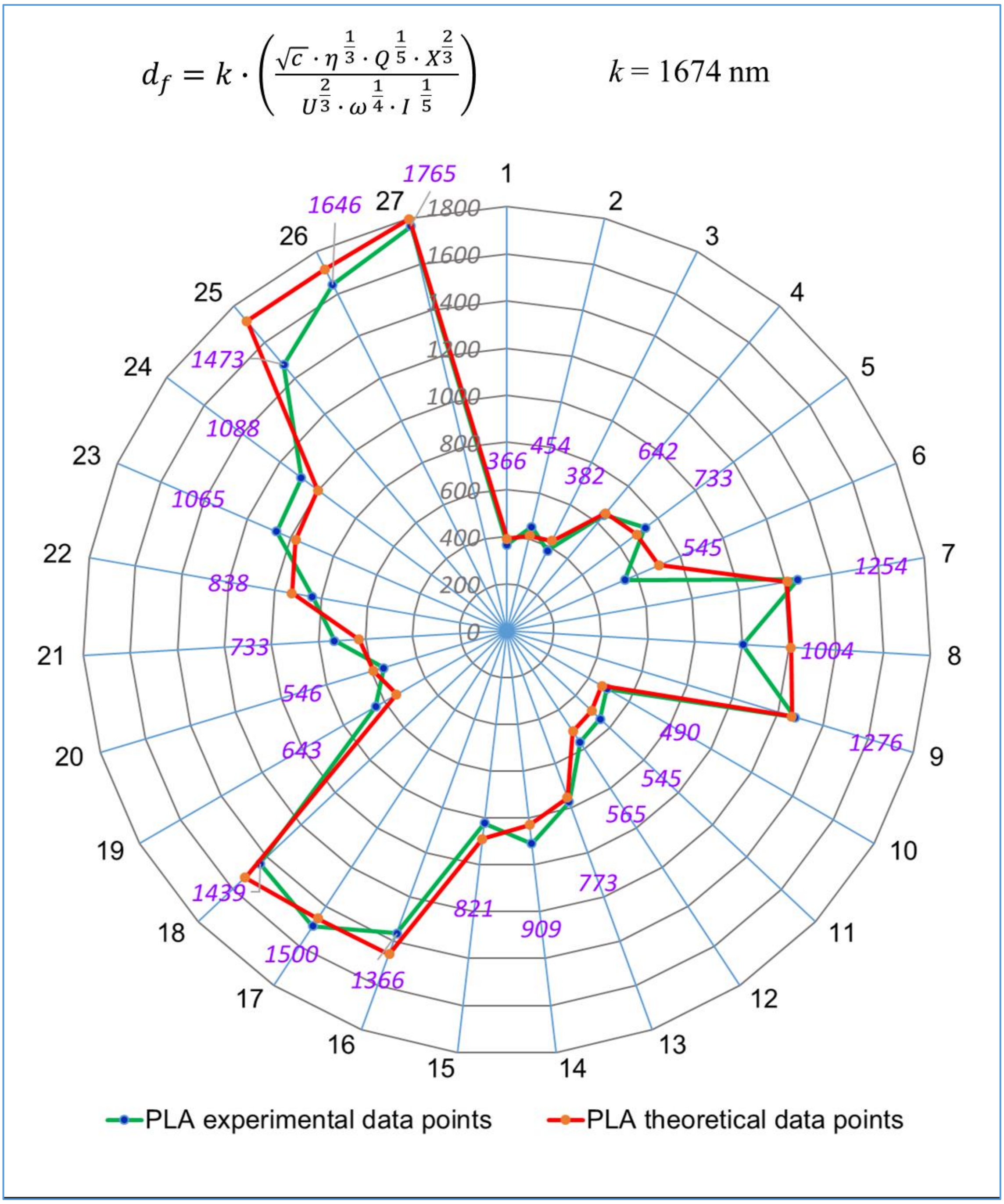

Figure S4. Theoretical and experimental diameter of electrospun fibers of PLA using emperical scaling laws (Eq.4) of various processing paramters.

S6. Determining the conductivity of spinning solutions using conductivity meter

PLA-PANi:CSA spinning solutions were prepared using mixture of chloroform (CF), dimethyl formamide (DMF) and ethanol (EtOH) with 4:0.3:0.7 ratio, respectively. To homogenize the solutions, they were kept on stirring for 12 h. Storage time of solutions was always less than 24 h. An electrical conductivity meter (SevenCompact Cond meter S230,

Mettler Toledo) was used to measure the conductivity of PLA-PANi:CSA spinning solutions at lab temperature (22°C). The conductivities of spinning solutions are stated in **Table S3**.

Table S3. Conductivity of spinning solutions varying composition of filler PANi and matrix polymer PLA in their solvents.

Filler Concentration (%)	Matrix concentration (%)	Solution conductivity (μS/cm)
0	100	0.013
1.8	98.2	2.84
3.5	96.5	3.48
5.4	94.6	4.67
9.8	90.2	5.45
16.6	83.4	6.6
21.1	78.9	7.14
27.5	72.5	7.3
36.4	63.6	7.7

S7. Determining the kinematic viscosity of spinning solutions using Ubbelohde viscometer

For the measurement of the kinematic viscosity, approximately 30 ml of each solution was filled in the Ubbelohde viscometer (Type capillary II_c, Internal diameter = 1.50 mm, SI Analytics, Weilheim, Germany) and stored in a water bath at 22 °C. Before starting the measurements, the viscometer containing the solutions was kept in the water bath for at least 5 minutes to reach the required temperature. Viscosity was measured using the corrected flow time multiplied by the viscometer constant gives the kinematic viscosity directly using **Equation S2** [4].

$$\eta = k(t - y) \quad \text{(S2)}$$

where, η is kinematic viscosity, k is the vicometer constant, t is average flow time through capillary and y is kinetic energy correction. The kinematic viscosity of spinning solutions are stated in **Table S4**.

Table S4. Kinematic viscosity of of spinning solutions varying composition of filler PANi and matrix polymer PLA in their solvents.

Filler Concentration	Matrix concentration	Kinematic viscosity
(%)	(%)	(mm^2/s)
0	100	297
1.8	98.2	282.32
3.5	96.5	266
5.4	94.6	254.68
9.8	90.2	224.43
16.6	83.4	185.18
21.1	78.9	157.2
27.5	72.5	126.6
36.4	63.6	76.59

S8. SEM statistical analysis

Examination of the electrospun fibers was performed by scanning electron microscopy (SEM) (AURIGA CrossBeam; Carl Zeiss Microscopy GmbH, Oberkochen, Germany) using an accelerating voltage of 5 kV. Prior to microscopy, the samples were coated with a 7.5 nm gold layer using a sputter coater (Q150T Turbo-pumped Sputter Coater, Quorum Technologies Inc., Guelph, ON, CA). The SEM images were further processed with Jmicro-Vision1.2.7 software to determine fiber diameter and orientation angle statistically, considering at least 100 measurements per investigated sample. The SEM images of PLA-PANi:CSA fibers were analyzed with JMicroVision software. JMicroVision can read/analyze images in many formats such GIF, TIF, DICOM, PEG, BMP, JPEG and FITS. In the present study TIF images were used. JMicroVision can calculate area and pixel value statistics of user defined selections. Firstly, pixel values of the images were converted into nanometer unit using the scale factor. Before using this command, the straight line selection tool was used to make a line selection that corresponded to known distance.

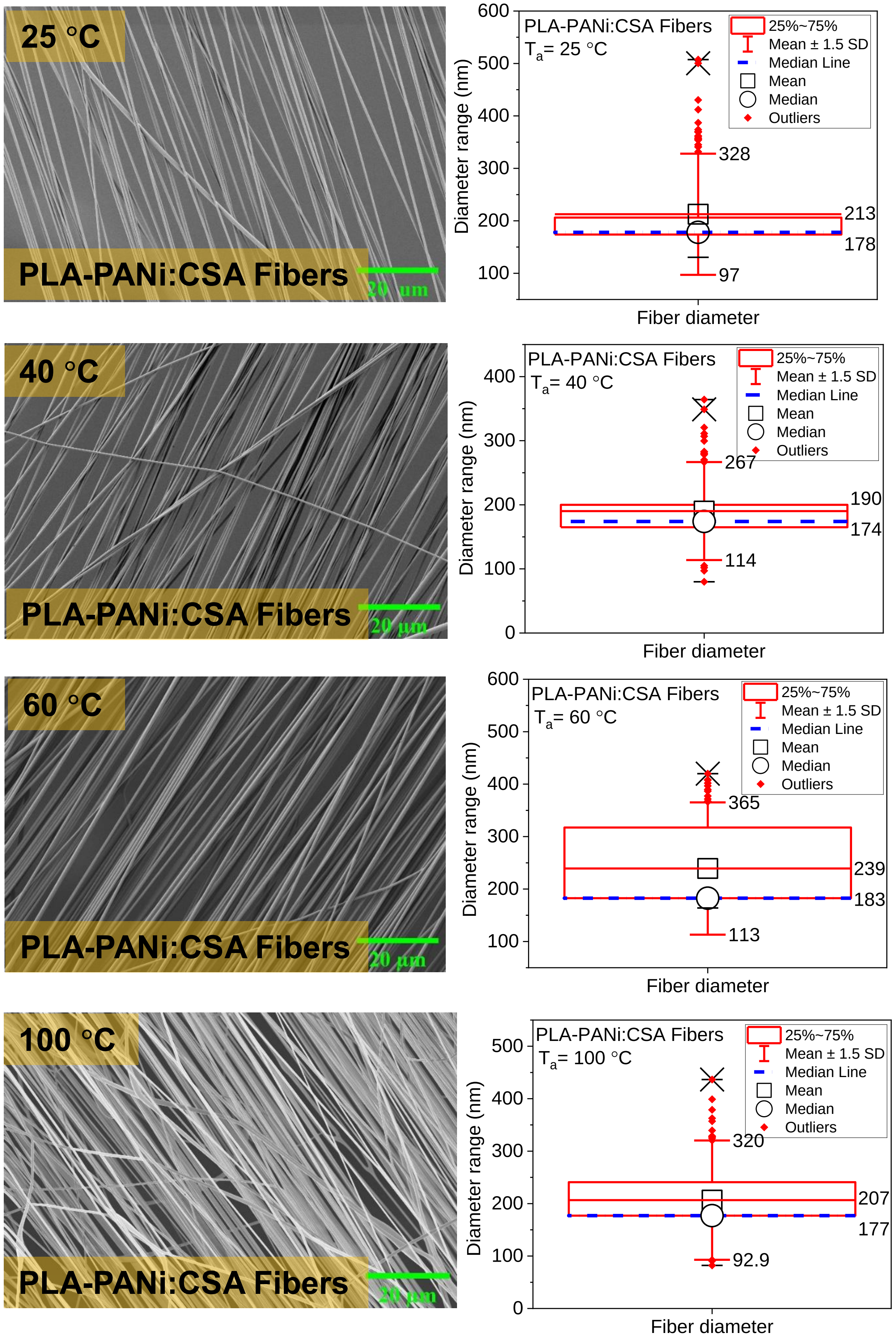

Figure S5. SEM images and corresponding box-plots for annealing temperature (T_a) 25, 40, 60 and 100°C, respectively.

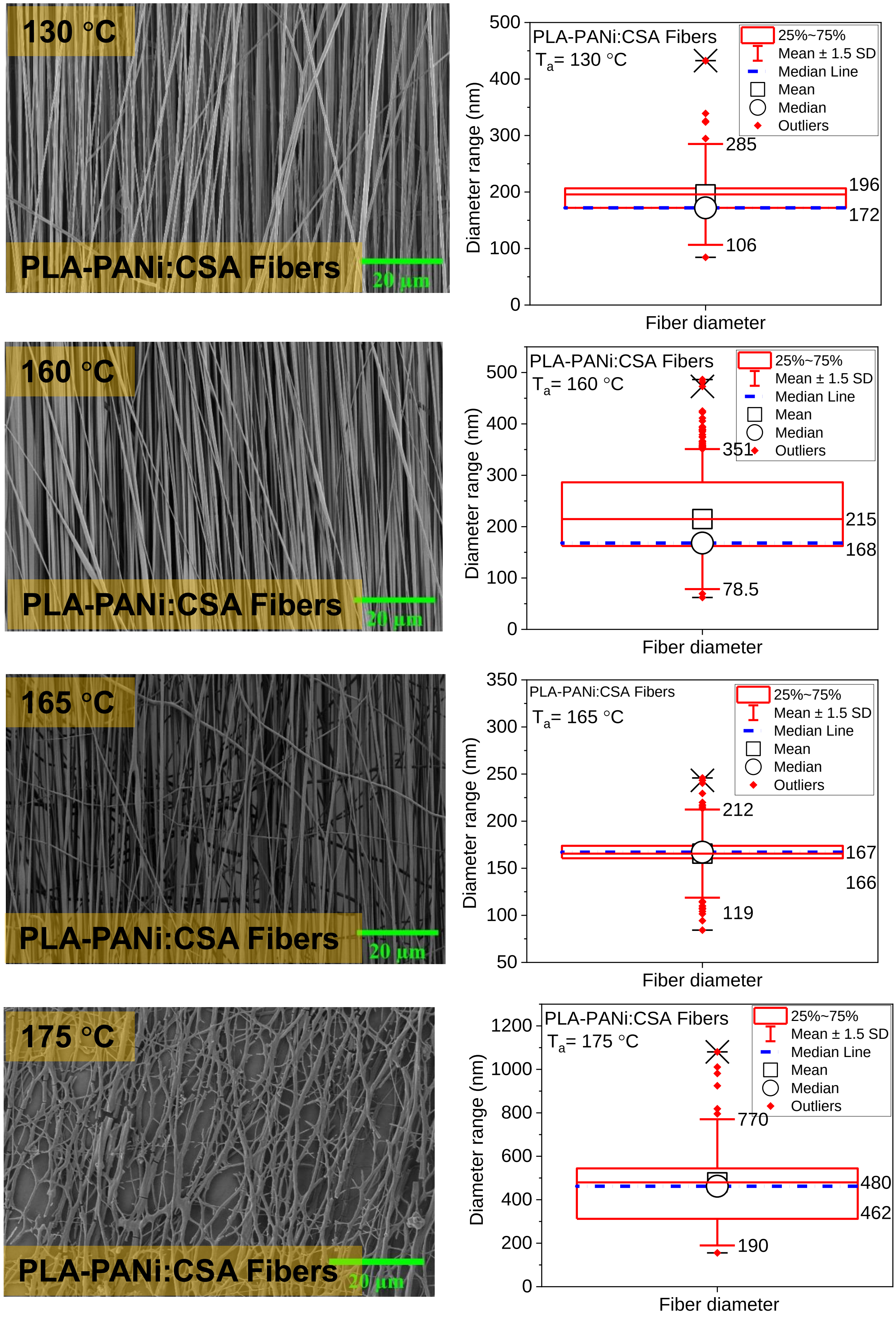

Figure S6. SEM images and corresponding box-plots for annealing temperature (T_a) 130, 160, 165 and 175°C, respectively.

The SEM images and corresponding box-plot analysis for median and mean fiber diameter at each annealing temperature (T_a) of 25, 40, 60, 100, 130, 160, 165 and 175°C are shown in **Figure S5** and **S6**, respectively. Box plots displaying the diameter size distribution of aligned fibers. The box outline is the interquartile range, the square and circle inside the box are the mean and median data points, the lines inside the box are the mean and median data locations, the whiskers display the upper inner and lower inner fence values. Above and below the whiskers: (−) maximum / minimum data point and (×) 99th percentile.

S9. TEM analysis

For the TEM sample preparation, scaffolds containing aligned fibers were fixed in Ito's fixative (Carl Roth, Karlsruhe, Germany). Semi-thin sagittal sections were created with a microtome (Ultracut E; Reichert Jung, Vienna, Austria). Transmission electron microscopy (TEM) imaging was performed using a JEM-1400 Plus (JEOL GmbH, Freising, Germany).

S10. Development of procedure and derivation of equations for calculating volume fraction of filler (φ) and density (ρ_c) of solid electrospun conductive nanofibrous biocomposites (CNBs)

The procedure for calculating volume fraction of filler (φ) and density (ρ_c) of CNBs is developed in this study. The densities of individual polymer matrix, filler and solvents are stated in **Table S1**. As the spinning solution containing the mixture of filler, matrix and solvent with different proportion of their respective components, the densities of filler-mixture (ρ_f), solvent-mixture (ρ_s), matrix-mixture (ρ_m), filler-solution (ρ_{fs}) and matrix-solution (ρ_{ms}) are calculated step by step by a rule of mixture [5];

$$\rho_f = \frac{1}{\frac{MF_{f1}}{\rho_{f1}} + \frac{MF_{f2}}{\rho_{f2}}} \quad \text{(S3)}$$

$$\rho_s = \frac{1}{\frac{MF_{S1}}{\rho_{S1}} + \frac{MF_{S2}}{\rho_{S2}}} \quad \text{(S4)}$$

$$\rho_m = \frac{1}{\frac{MF_{m1}}{\rho_{m1}} + \frac{MF_{m2}}{\rho_{m2}}} \quad \text{(S5)}$$

where; *MF* is mass fraction and the subscripts f_1 and f_2, S_1 and S_2 and m_1 and m_2 show the two components of filler-mixture, solvent-mixture and matrix-mixture, respectively.

As filler-solution and matrix-solution with their respective solvent systems were prepared in weight/weight percentage (w/w %) concentrations with **Equation S6** and **S7**, respectively

$$\frac{W}{W}\ \% = \frac{M_f}{M_{fs}} \cdot 100 = W_{fs} \quad \text{(S6)}$$

$$\frac{W}{W}\ \% = \frac{M_m}{M_{ms}} \cdot 100 = W_{ms} \quad \text{(S7)}$$

where; M_f and M_m are masses of filler and matrix in their respective masses of filler-solution (M_{fs}) and matrix-solution (M_{ms}), respectively. For simplicity, W_{fs} and W_{ms} are considered equivalent terms to overall masses of filler-solution and matrix-solution, respectively.

The densities of filler-solution (ρ_{fs}) and matrix-solution (ρ_{ms}) are determined using **Equation S8** and **S9**, respectively.

$$\rho_{fs} = \frac{1}{\frac{MF_f}{\rho_m} + \frac{MF_s}{\rho_s}} \quad \text{(S8)}$$

$$\rho_{ms} = \frac{1}{\frac{MF_m}{\rho_m} + \frac{MF_s}{\rho_s}} \quad \text{(S9)}$$

where; MF_m, MF_f and MF_s are mass fractions of matrix, filler and solvent, respectively.

To convert w/w % concentrations to v/v % (volume/volume percentage) concentrations, **Equation S6** and **S7** were divided by the densities of their respective species by using **Equation S8** and **S9**, respectively.

$$\frac{V}{V}\ \% = \frac{M_f}{M_{fs}} \cdot \frac{\rho_{fs}}{\rho_f} \cdot 100 = V_{fs} \quad \text{(S10)}$$

$$\frac{V}{V}\ \% = \frac{M_m}{M_{ms}} \cdot \frac{\rho_{ms}}{\rho_m} \cdot 100 = V_{ms} \quad \text{(S11)}$$

where; V_{ms} and V_{fs} are the volumes of matrix-solution and filler-solution, respectively.

To prepare matrix-filler solution for electrospinning, *volume x* of filler-solution (xV_{fs}) and *volume y* of matrix-solution (yV_{ms}) were mixed as shown in schematic **Figure S7**.

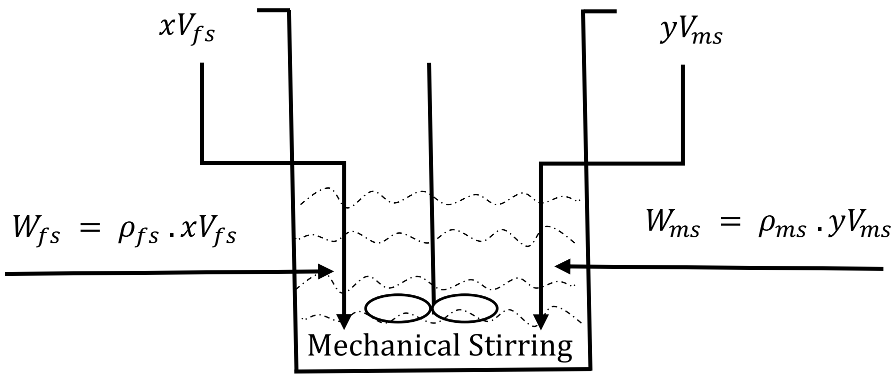

Figure S7. Schematic of mixing of *volume x* of filler-solution (xV_{fs}) and *volume y* of matrix-solution (yV_{ms}) with densities ρ_{fs} and ρ_{ms}, respectively.

When filler-matrix solution was spun in electrospinning, the solvent evaporated and solid composite fibers of filler and matrix were obtained. The volume fraction of filler ($VF_f = \varphi$) in dried solid CNBs was calculated as;

$$\varphi = VF_f = \frac{xV_{fs}}{xV_{fs}+yV_{ms}} \quad \text{(S12)}$$

The density of solid CNBs (ρ_c) was determined by rule of mixture;

$$\rho_c = \frac{1}{\left(\frac{xV_{fs} \cdot \rho_{fs}}{xV_{fs} \cdot \rho_{fs}+yV_{ms} \cdot \rho_{ms}}\right) \cdot \frac{1}{\rho_f} + \left(\frac{yV_{ms} \cdot \rho_{ms}}{xV_{fs} \cdot \rho_{fs}+yV_{ms} \cdot \rho_{ms}}\right) \cdot \frac{1}{\rho_m}} \quad \text{(S13)}$$

To simplify the **Equation S13**;

$$WF_f = \frac{xV_{fs} \cdot \rho_{fs}}{xV_{fs} \cdot \rho_{fs}+yV_{ms} \cdot \rho_{ms}} \quad \text{(S14)}$$

$$WF_m = \frac{yV_{ms} \cdot \rho_{ms}}{xV_{fs} \cdot \rho_{fs}+yV_{ms} \cdot \rho_{ms}} \quad \text{(S15)}$$

where; WF_f and WF_m are the weight fractions of filler and matrix, respectively and a simplified form of **Equation S13 is**;

$$\rho_c = \frac{1}{\frac{WF_f}{\rho_f} + \frac{WF_m}{\rho_m}} \quad \text{(S16)}$$

S11. Identifying electrical conductivity of electrospun fibers

The highly aligned fibers were collected on microscopic glass slides (GS) (**Figure S1f**) for the measurement of resistance *R* using an ohmmeter (Keithley 2400) (**Figure S1g** and **S1h**). All samples of conductive fibers (CNBs) were annealed isothermally in a vacuum oven (**Figure S1i**), at their respective temperatures for 24 hours and subsequently quenched to room temperature. Two opposite sides of the GS, were coated with silver-ink to ensure metallic contact with the measuring electrodes.

An equation is derived to calculate the conductivity σ of CNBs. First consider a single fiber of length L and radius r, which has cross-sectional area $A_e = \pi r^2$ (as shown in schematic **Figure S1j**). The fiber has uniform radius along its whole length.

At constant temperature, the resistance of a single fiber R_e;

$$R_e = \frac{\mu_e \cdot L}{\pi r^2} \tag{S17}$$

where; μ_e is resistivity of a single fiber. For n fibers on GS follows;

$$R = \frac{\mu \cdot L}{n\pi r^2} \tag{S18}$$

where; R and μ are total resistance and resistivity of n fibers, respectively.

As volume of n cylindrical fibers on GS; $V = n\pi r^2 L$

$$n\pi r^2 L = \frac{W_c}{\rho_c} \tag{S19}$$

where; W_c and ρ_c are the final weight and density of dried CNBs.

Combining and re-arranging **Equations S18** and **19**;

$$R = \frac{\mu \cdot L^2 \cdot \rho_c}{W_c} \tag{S20}$$

Since conductivity σ is the reciprocal of the resistivity μ;

$$\sigma = \frac{1}{\mu} = \frac{L^2 \cdot \rho_c}{R \cdot W_c} \tag{S21}$$

Using **Equation S21**, the electrical conductivity σ of CNBs can be calculated using the value of R, which was measured using a two-point probe method for a constant voltage (*1V*) at room

temperature, W_c that was measured before applying the silver-ink paste, L for all fibers that is equal to the length of a single fiber and ρ_c (density of dried CNBs) that was calculated from **Equation S16**.

S12. Annealing process for conductive fibers

All samples of oriented electrospun conductive nanofibrous biocomposites (CNBs) were annealed isothermally in a vacuum oven at temperatures of 25, 40, 60, 100, 130, 160, 170 and 180°C with time intervals of 30, 60, 90, 120, 360, 720, 1080 and 1440 min, respectively. All CNBs samples were collected on glass slides. First, the vacuum oven was turned on and heated up to the required temperature and after reaching stable heating conditions, the samples were put into the airtight oven and the vacuum turned on to avoid degradation due to air during heating. After each annealing temperature and time, the sample was taken out of the oven to allow cooling to room temperature.

S13. Experimental procedure for complex viscosity measurement using rheometer

Pure PLA pellets were pre-heated in a vacuum oven at 35°C for 4 h to remove residual moisture content. The dried PLA pellets were pressed into round geometry (25 mm diameter and 2 mm height) using a hot press at 190 °C at 10 bar pressure. To achieve bubble free samples (round plates) and exclude degradation of the material, the whole pressing process was performed under vacuum. Shear experiments were carried out with the DHR 3 rheometer from TA Instruments (USA) with a plate-plate geometry (25 mm diameter, 1800 μm gap). To prevent polymer degradation, a nitrogen atmosphere was applied during the experiments. Frequency sweeps were performed at four temperatures (150°C, 160°C, 170°C and 180°C) in a range of 0.1 – 628 rad s^{-1}. Previously, oscillating shear experiments were performed to test the linear viscoelastic regime (amplitude sweeps). On varying the amplitude from 0.001 to 100%, the optimal choice was revealed at 3%.

S14. DSC calculations and crystallinity measurements

The crystallinity of the oriented electrospun conductive nanofibrous biocomposites (CNBs) of PLA-PANi:CSA was determined using a differential scanning calorimeter (DSC) (Model DSC Q 2000, TA Instruments). The recommended amount (5-10 mg) of sample material was sealed in aluminium pans, and the scans were acquired between 20-200 °C at a scan rate of 10 °C /min in dry nitrogen atmosphere. DSC thermograms of pure un-processed PLA pellets, thermograms of electrospun fibers of pure PLA and thermograms of annealed PLA-PANi:CSA fibers with 10% filler concentration (φ = 10%) are shown in **Figure S8**.

The absolute degree of crystallinity (χ_c) can be calculated with **Equation S22** [6];

$$\chi_c(\%) = \frac{\Delta H_f}{\Delta H_f^{\circ}} \, . 100 \qquad \text{(S22)}$$

Where; ΔH_f, and ΔH_f° are the apparent heat of fusion per gram of the sample and the thermodynamic heat of fusion per gram of 100% crystalline polymer, respectively. The value of ΔH_f° for PLA is 93 J/g [6].

To determine the crystallinity of the electrospun fibers, the extra heat released during heating (i.e., enthalpy of cold crystallization; ΔH_c) was subtracted from the total endothermic heat flow during melting of all crystallites. Thus, **Equation S23** [6] gives;

$$\chi_c(\%) = \frac{\Delta H_f - \Delta H_c}{\Delta H_f^{\circ}} \, . 100 \qquad \text{(S23)}$$

The melting temperature (T_m), ΔH_f, and ΔH_c were determined from DSC thermograms. Parameters T_m and ΔH_f were taken as the peak temperature and the area of the melting endotherm, respectively. The DSC thermograms (**Figure S8**) and table (**Table S5**) depict the thermodynamic characteristics of glass transition temperature (T_g), crystallization temperature (T_c), T_m, ΔH_c, ΔH_f°, ΔH_f and χ_c for all samples.

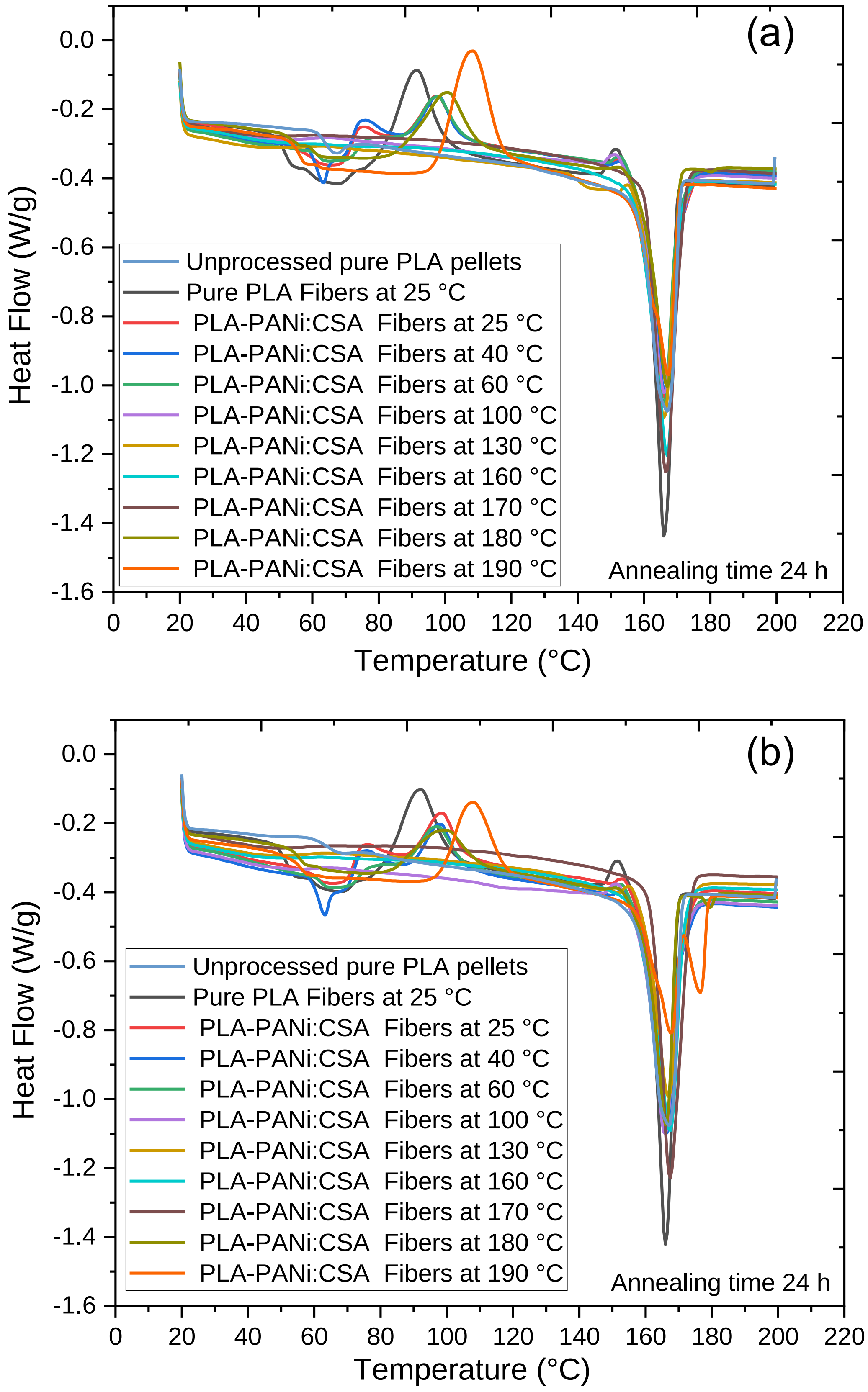

Figure S8. DSC thermograms for pure PLA pellets (un-processed), pure PLA fibers and annealed PLA-PANi:CSA fibers with $\varphi_{PANi} \approx 10\%$. Panels a and b show first and second time measurements, as replicates of the same material compositions, respectively.

Table S5. DSC data after first heating cycle with replicates of the same material compositions. The value of ΔH_f° for PLA (93 J/g) was taken from the literature [6].

Samples with their annealing temperatures	T_g (°C)	T_c (°C)	T_m (°C)	ΔH_c (J/g)	ΔH_f° (J/g)	ΔH_f (J/g)	χ_c (%)
1-Pure PLA pellets	62	-	167	25.71	35.13	93	37.7
2-Pure PLA pellets	61.8	-	167	22.74	36.67	93	39.4
1-Pure PLA fibers	49.5	91.1	166	7.13	48.4	93	24.4
2-Pure PLA fibers	49.1	91.7	166	6.58	43.5	93	22.3
1-PLA-PANi:CSA fibers (25 °C)	65.1	97.8	166.4	5.64	27.12	93	21.5
2-PLA-PANi:CSA fibers (25 °C)	58.1	98.6	166.3	6.37	23.98	93	18.7
1-PLA-PANi:CSA fibers (40 °C)	59.3	98.1	166.4	3.24	27.12	93	23.1
2-PLA-PANi:CSA fibers (40 °C)	59.5	98.1	166.2	4.45	27.14	93	22.3
1-PLA-PANi:CSA fibers (60 °C)	60.9	98.1	166	25.71	29.45	93	28.2
2-PLA-PANi:CSA fibers (60 °C)	60.9	97.2	166.1	22.74	26.78	93	24.0
1-PLA-PANi:CSA fibers (100 °C)	-	-	166.1	-	28.72	93	30.9
2-PLA-PANi:CSA fibers (100 °C)	-	-	165.7	-	31.13	93	33.5
1-PLA-PANi:CSA fibers (130 °C)	-	-	166	-	37.12	93	39.9
2-PLA-PANi:CSA fibers (130 °C)	-	-	166.8	-	32.65	93	35.1
1-PLA-PANi:CSA fibers (160 °C)	-	-	166.9	-	38.53	93	41.4
2-PLA-PANi:CSA fibers (160 °C)	-	-	167.4	-	36.81	93	39.6
1-PLA-PANi:CSA fibers (170 °C)	-	-	166.7	-	37.76	93	40.6
2-PLA-PANi:CSA fibers (170 °C)	-	-	166.3	-	37.95	93	40.8
1-PLA-PANi:CSA fibers (180 °C)	58.1	99.6	167	11.18	27.01	93	17.0
2-PLA-PANi:CSA fibers (180 °C)	53.9	98.6	166.1	10.61	27.65	93	18.3
1-PLA-PANi:CSA fibers (190 °C)	54.2	107.5	167.2	23.43	28.69	93	5.66
2-PLA-PANi:CSA fibers (190 °C)	53.7	107.4	167.6	17.15	24.26	93	7.65

S15. Identifying elasticity of electrospun fibers

The single fiber tensile test is performed to determine the mechanical properties of CNBs. The schematic of the mechanical testing process and photo of the Single-fiber tensile testing machine (Vibrodyn 400, Lenzing instruments GmbH & Co. KG, Germany) are shown in **Figure S1k-o**. **Figures S1p** and **S1q** show a fiber bundle on the SEM holder and SEM image

of a fiber bundle, respectively. Under the conditions of 200 mg load and 10 mm clamping length within the VPN program, the fiber bundle is stretched at a speed of 10 mm/min until it breaks, providing its force-strain curve. Three replicates are investigated for each kind of fiber. Before the tensile testing, the tensile positions are marked on each fiber bundle. After testing, the fiber bundle is cut off along the marks. The weight of each fiber bundle (within the marked length) is measured.

An equation is derived to calculate the elastic Young's modulus (*E*) of CNBs. The highly aligned fibers are collected on the glass slide (GS) and rolled into a bundle of parallel fibers. Considering a bundle of *n* fibers with length *L* and radius *r*, which has cross-sectional area $A = n\pi r^2$, under optimal conditions, the Young's modulus of a fiber, *E*;

$$E = \frac{\sigma}{\varepsilon} \tag{S24a}$$

$$\sigma = \frac{F}{A} \tag{S24b}$$

$$\varepsilon = \frac{\Delta L}{L} \tag{S24c}$$

where; σ, ε, F, A, ΔL and L are stress, strain, force, cross-sectional area, change in length and length of sample, respectively.

As volume of a bundle of fibers; $V = n\pi r^2 L$

$$V = \frac{W_c}{\rho_c} \tag{S25a}$$

$$V = A \cdot L \tag{S25b}$$

where; W_c and ρ_c are the final weight (within the red marks) and density (as calculated using Equation S16) of bundle fiber, respectively.

Combining and re-arranging **Equations S24a**, **S24b**, **S24c**, **S25a** and **S25b**;

$$E = \frac{F \cdot L}{\Delta L} \cdot \frac{\rho_c \cdot L}{W_c} \tag{S26a}$$

$$E = k \cdot \frac{\rho_c \cdot L}{W_c} \tag{S26b}$$

where; $k = \frac{F \cdot L}{\Delta L}$ is the slope of the force-strain curve in the linear elastic region.

The Young’s modulus (E) of a bundle of fibers can be determined using **Equation S26**.

References

(1) Schubert, D. W. Revealing Novel Power Laws and Quantization in Electrospinning Considering Jet Splitting—Toward Predicting Fiber Diameter and Its Distribution. *Macromol. Theory Simul.* **2019**, *28* (4), 1900006. DOI: 10.1002/mats.201900006.
(2) Schubert, D. W.; Allen, V.; Dippel, U. Revealing Novel Power Laws and Quantization in Electrospinning Considering Jet Splitting—Toward Predicting Fiber Diameter and Its Distribution Part II Experimental. *Advanced Engineering Materials* **2021.** DOI: 10.1002/adem.202001161.
(3) Munawar, M. A.; Schubert, D. W. Modeling and optimization of diameter of highly oriented electrospun nanofibers of biopolymer.
(4) *DIN 51562-1:1999-01, Viskosimetrie_- Messung der kinematischen Viskosität mit dem Ubbelohde-Viskosimeter_- Teil_1: Bauform und Durchführung der Messung*; Beuth Verlag GmbH, Berlin.
(5) Tam, D.; Ruan, S.; Gao, P.; Yu, T. 10 - High-performance ballistic protection using polymer nanocomposites. In *Advances in Military Textiles and Personal Equipment : Woodhead Publishing Series in Textiles*; Sparks, E., Ed.; Woodhead Publishing, 2012; pp 213–237. DOI: 10.1533/9780857095572.2.213.
(6) Srithep, Y.; Nealey, P.; Turng, L.-S. Effects of annealing time and temperature on the crystallinity and heat resistance behavior of injection-molded poly(lactic acid). *Polym Eng Sci* **2013**, *53* (3), 580–588. DOI: 10.1002/pen.23304.
